# Functional signatures of oral dysbiosis during periodontitis progression revealed by microbial metatranscriptome analysis

**DOI:** 10.1186/s13073-015-0153-3

**Published:** 2015-04-27

**Authors:** Susan Yost, Ana E Duran-Pinedo, Ricardo Teles, Keerthana Krishnan, Jorge Frias-Lopez

**Affiliations:** Forsyth Institute, 245 First Street, Cambridge, Massachusetts 02142 USA; Harvard School of Dental Medicine, 188 Longwood Ave, Boston, MA 02115 USA; University of North Carolina Chapel Hill, School of Dentistry, Chapel Hill, NC 27599-7450 USA

## Abstract

**Background:**

Periodontitis is a polymicrobial biofilm-induced inflammatory disease that affects 743 million people worldwide. The current model to explain periodontitis progression proposes that changes in the relative abundance of members of the oral microbiome lead to dysbiosis in the host-microbiome crosstalk and then to inflammation and bone loss. Using combined metagenome/metatranscriptome analysis of the subgingival microbiome in progressing and non-progressing sites, we have characterized the distinct molecular signatures of periodontitis progression.

**Methods:**

Metatranscriptome analysis was conducted on samples from subgingival biofilms from progressing and stable sites from periodontitis patients. Community-wide expression profiles were obtained using Next Generation Sequencing (Illumina). Sequences were aligned using ‘bowtie2’ against a constructed oral microbiome database. Differential expression analysis was performed using the non-parametric algorithm implemented on the R package ‘NOISeqBio’. We summarized global functional activities of the oral microbial community by set enrichment analysis based on the Gene Ontology (GO) orthology.

**Results:**

Gene ontology enrichment analysis showed an over-representation in the baseline of active sites of terms related to cell motility, lipid A and peptidoglycan biosynthesis, and transport of iron, potassium, and amino acids. Periodontal pathogens (*Tannerella forsythia* and *Porphyromonas gingivalis*) upregulated different TonB-dependent receptors, peptidases, proteases, aerotolerance genes, iron transport genes, hemolysins, and CRISPR-associated genes. Surprisingly, organisms that have not been usually associated with the disease (*Streptococcus oralis*, *Streptococcus mutans*, *Streptococcus intermedius*, *Streptococcus mitis*, *Veillonella parvula,* and *Pseudomonas fluorenscens*) were highly active transcribing putative virulence factors. We detected patterns of activities associated with progression of clinical traits. Among those we found that the profiles of expression of cobalamin biosynthesis, proteolysis, and potassium transport were associated with the evolution towards disease.

**Conclusions:**

We identified metabolic changes in the microbial community associated with the initial stages of dysbiosis. Regardless of the overall composition of the community, certain metabolic signatures are consistent with disease progression. Our results suggest that the whole community, and not just a handful of oral pathogens, is responsible for an increase in virulence that leads to progression.

**Trial registration:**

NCT01489839, 6 December 2011.

**Electronic supplementary material:**

The online version of this article (doi:10.1186/s13073-015-0153-3) contains supplementary material, which is available to authorized users.

## Background

Much has been learned about the diversity and distribution of oral associated microbial communities, but we still know little about the biology of the microbiome, how it interacts with the host, and how the host responds to its resident microbiota. The oral cavity offers a unique opportunity to study how microbial communities have an influence on the health status of their human host. Imbalances of the oral microbiota, also referred to as microbial dysbiosis, lead to a series of different oral diseases. These dysbiotic microbial communities exhibit synergistic interactions for enhanced protection from host defenses, nutrient acquisition, and persistence in an inflammatory environment [1,2]. Periodontitis is an oral polymicrobial disease caused by the coordinated action of a complex microbial community, which results in inflammation and destruction of the periodontium in susceptible hosts. Periodontal disease is the sixth most prevalent health condition in the world affecting 743 million people worldwide [3]. It occurs in moderate form in 30% to 50% of American adults and in severe form in 10% of the population and is responsible for half of all tooth loss in adults [4,5]. In addition, recent studies have suggested that periodontal diseases can influence the risk for certain systemic conditions such as cardiovascular diseases, diabetes, respiratory diseases, and can affect reproductive outcomes [6,7].

Using checkerboard, a DNA-DNA hybridization technique, periodontitis-associated taxa have been cataloged into groups or complexes, representing bacterial consortia that appear to occur together and that are associated with various stages of disease [7]. The red complex, which appears later in biofilm development, comprises three species that are considered to be the major periodontal pathogens: *Porphyromonas gingivalis, Treponema denticola,* and *Tannerella forsythia* [7,8]. Another important group of organisms that has been associated with chronic periodontitis is the orange complex constituted by: *Fusobacterium nucleatum, Prevotella intermedia, Prevotella nigrescens, Parvimonas micra*, *Streptococcus constellatus, Eubacterium nodatum, Campylobacter showae, Campylobacter gracilis, and Campylobacter rectus.* Similar to the red complex, all species in the orange complex have been shown to have a significant association with increasing pocket depth [7,9] and reciprocal interactions between both have been proposed [9]. In more recent studies using 454 pyrosequencing to characterize healthy and periodontitis microbial communities [10,11], the overall picture of bacterial associations with health and disease agree with the initial descriptions of the different oral microbial complexes.

One key question to be answered regarding the pathogenesis of polymicrobial diseases is what the molecular mechanisms that lead to dysbiosis are. In the case of periodontitis, why in some cases teeth with clinical symptoms of periodontitis progress leading to tooth loss (if untreated) and in some other cases the progression of the disease stops despite lack of treatment [12]. There have been a large number of attempts to identify reliable markers that would distinguish between progressing and non-progressing sites. These include genetic markers [13,14], protein activity [15,16], cytokines [17,18], and bacterial [19,20] and clinical markers [19]. However, none of these associations explain why progression occurs.

Current models of periodontal disease progression posit that tissue destruction occurs through periods of acute exacerbations (activity) followed by periods of remission [21,22]. It has been postulated that changes in the composition of subgingival biofilms could explain these periods of disease activity. In fact, a few papers have found differences in the levels of subgingival species when comparing progressing and non-progressing sites using cultivation techniques [23] and molecular approaches such as real-time PCR [20]. However, these studies also demonstrated considerable overlap in the composition of the microbial communities associated with active and non-progressing lesions, suggesting that the difference in the periodontal status of the sites could not be explained solely by the reported differences in the subgingival microbial composition.

Cataloging the activities of each bacterial species in a community may provide more insight into pathogenesis than simple enumeration of that community’s gene content. This is because the community functions as a system, and its activities and interactions control the fate of the microbiome.

The goal of the present study was to characterize *in situ* gene expression patterns of the whole oral microbiome during periodontitis progression to identify early steps in dysbiosis that could answer the question of why only certain teeth progress to disease while others do not.

## Methods

### Ethics statement

Written informed consent was obtained from all participants in this study. Recruitment of study participants was performed according to protocol (ClinicalTrials.gov ID NCT01489839) approved by Forsyth Institutional Review Board ID #11-09 (Approval Date 8 August 2014) in Cambridge, MA, USA. All subjects provided written signed informed consent prior to participation.

### Power calculation

In order to assess the sample size required we used the R package RNASeqPower [24]. We first estimated the average coverage using samtools depth command from the samtools package [25]. According to this analysis, with an FDR of 0.05 and a target effect size of 2, we need eight subjects in each group to have a power of 0.9.

### Study design, subject population, and sample collection

The subjects in the present study were recruited as part of a multi-center clinical trial to determine biomarkers of periodontal disease progression (Clinical Trials.gov ID NCT01489839). Under this ongoing study, subjects are monitored clinically for a period of up to 1 year every 2 months in order to detect periodontal sites and subjects with periodontal disease progression. Subgingival microbial samples are collected from up to 32 sites per subject per visit. The Institutional Review Board at The Forsyth Institute approved all aspects of the study protocol. The study was described thoroughly to all subjects prior to obtaining informed consent. Inclusion criteria were: age >24 years; ≥20 natural teeth (excluding third molars); at least four teeth with at least one site of pocket depth (PD) of 5 mm or more and concomitant clinical attachment loss (CAL) greater than or equal to 2 mm; radiographic evidence of mesial or distal alveolar bone loss around at least two of the affected teeth; and in good general health (Table 1). Exclusion criteria were: current cigarette smokers; pregnant or nursing; received antibiotic or periodontal therapy in the previous 6 months; any systemic condition potentially affecting the course of periodontal disease (for example, diabetes or AIDS); chronic use of non-steroidal anti-inflammatory drugs; or any condition requiring antibiotic coverage for dental procedures. Disease progression at a site was defined by an increase in CAL ≥2 mm at any follow-up visit compared with baseline. Stable sites were characterized by no change in CAL >1 mm from baseline. Eight stable sites and eight progressing sites from the nine subjects were analyzed (Table 2). We collected one stable site and one progressing site, both at baseline and at the endpoint of analysis. For seven of the nine subjects, both progressing and stable sites matched the initial baselines. Samples were processed as described below.

**Table 1:** **Clinical and demographic characteristics of study subjects**

**Subject**	**Age (years)**	**Mean PD (mm)**	**Mean CAL (mm)**	**Sites with PD ≥5 mm (%)**
1	53	2.1	1.4	7%
2	32	2.5	1.8	15%
3	54	3.0	3.0	17%
4	50	3.9	3.3	41%
5	59	3.1	3.3	21%
6	42	2.1	2.5	3%
7	75	1.9	3.4	10%
8	66	2.0	2.2	7%
9	63	1.9	1.0	4%

CAL: clinical attachment loss; PD: pocket depth.

**Table 2:** **Clinical characteristics of progressing and stable sites**

**Subject**	**Site** ^**a**^	**Visit (months)**	**PD (mm)**	**CAL (mm)**	**Progression (0/1)**
1	361	0	3.0	2.0	1
	361	2	5.0	4.0	
1	353	0	2.5	1.5	0
	353	2	3.0	2.0	
2	473	0	3.0	2.0	1
	473	2	5.0	4.0	
2	241	0	3.0	1.0	0
	241	2	3.0	2.0	
3	273	0	4.0	4.0	1
	273	2	8.5	8.0	
3	441	0	4.0	3.0	0
	441	2	3.0	2.0	
4	143	0	6.0	5.0	1
	143	2	8.0	7.0	
4	453	0	4.5	3.5	0
	453	2	5.0	4.0	
5	151	0	3.0	3.0	1
	151	2	5.0	5.5	
5	373	0	3.0	3.0	0
	373	2	3.0	3.0	
6	143	0	2.0	2.0	1
	143	4	4.0	4.0	
6	151	0	1.5	2.5	0
	151	2	1.0	2.0	
7	253	0	2.5	2.5	1
	253	4	5.0	5.0	
7	141	0	2.0	3.0	0
	141	2	3.0	3.5	
8	253	0	3.5	3.5	1
	253	2	5.5	5.5	
9	353	0	2.0	1.0	0
	353	2	2.5	1.5	

^a^First two digits indicate tooth number according to the FDI World Dental Federation two-digit notation; third digit indicates site position: 1 - mesio-buccal, 3 - disto-buccal.

CAL: clinical attachment loss; PD: pocket depth.

After removal of supragingival plaque, subgingival plaque samples were taken separately from the mesio-buccal and disto-buccal sites of pre-molars and first and second molars using individual sterile Gracey curettes and each sample placed in individual tubes containing 200 uL of RNAse-free TE buffer, immediately frozen and stored at -80°C until processed.

### Community DNA and RNA extraction

Cells were collected by centrifugation for 10 min at maximum speed in a microcentrifuge. A total of 600 μL of mirVana kit lysis/binding buffer and 300 μL of 0.1-mm zirconia-silica beads (BioSpec Products, Bartlesville, OK, USA) were added to the samples. The beads were treated with DEPC overnight and autoclaved. Samples were bead beaten for 1 min at maximum speed. DNA and RNA were extracted simultaneously following the protocol of *mir*Vana^TM^ Isolation kit for RNA and ToTALLY RNA^TM^ kit (Life Technologies) for DNA. Eukaryotic DNA was removed using the MolYsis® kit (Molzym GmbH & Co. KG, Bremen, Germany). MICROBioEnrich (Life Technologies) was used to remove eukaryotic RNA and MICROB*Express* (Life Technologies) to remove prokaryotic rRNA. All kits were used following the manufacturers’ instructions.

### DNA, RNA amplification, and Illumina sequencing

DNA amplification was performed using the Illustra GenomiPhi V2 amplification kit (GE Healthcare Life Sciences) according to the manufacturer’s instructions. RNA amplification was performed on total bacterial RNA using MessageAmp^TM^ II-Bacteria RNA amplification kit (Life Technologies) following the manufacturer’s instructions. Sequencing was performed at the Forsyth Institute. Illumina adapter-specific primers were used to amplify and selectively enrich for the cDNA generated from enriched mRNA. Quantified libraries were pooled and sequenced using the MiSeq v2, 2×150 cycle cartridge (Illumina). The Nextera XT kit was used to generate libraries from amplified DNA. Normalized libraries were pooled and sequenced using the 2×250 Miseq v2 cartridge.

### Selection of genomes in databases

Genomes of archaea and bacteria as well as their associated information were downloaded from the HOMD database server [26], the Pathosystems Resource Integration Center (PATRIC) ftp server [27,28], and the J. Craig Venter Institute [29]. A total of 524 genomes from 312 species of bacteria and two genomes from one archaea species were used in the analysis (Additional file 1: Table S7). Viral genomes were downloaded from NCBI [30].

### Short reads sequence alignment analysis

Low-quality sequences were removed from the query files. Fast clipper and fastq quality filter from the Fastx-toolkit [31] were used to remove short sequences with quality score >20 in >80% of the sequence. Cleaned files were then aligned against the bacterial/archaeal database using bowtie2. We generated a .gff file to map hits to different regions in the genomes of our database. Read counts from the SAM files were obtained using bedtools multicov from bedtools [32].

### Phylogenetic analysis of the metagenome and metatranscriptome

Counts from the DNA and RNA libraries were used to determine the phylogenetic composition of the respective libraries. We created a .gff file containing information on whole genomes that was used to assign hits to genomes. Abundance estimation at the species level was performed applying the Genome Abundance Similarity Correction (GASiC) proposed by Lindner and Renard to estimate true genome abundances via read alignment by considering reference genome similarities in a non-negative LASSO approach [33]. Estimated counts were normalized by frequency and log2 transformed before final analysis. To identify significant differences between communities under the different conditions studied we performed linear discriminant analysis (LDA) effect size (LEfSe) as proposed by Segata *et al.* [34], with default settings.

### Differential expression analysis

For assessing differential expression in genes within a specific species we normalized the transcript counts by the relative frequency of the species in the metagenome database. In the case of Gene Ontology (GO) term analysis, we did not normalize by relative abundance since we were treating the whole community as a single organism. To identify differentially expressed genes from the RNA libraries, we applied non-parametric tests to the normalized counts using NOISeqBIO function of the R package NOISeq default conditions (k = 0.5, lc = 1, replicates = ‘biological’ and rpkm normalization (rpkm option) using the threshold value for significance suggested by the authors of q = 0.95, which for the function NOISeqBio is equivalent to an FDR cutoff of 0.05 [35,36].

### GO enrichment analysis

To evaluate functional activities differentially represented in health or disease, we mapped the differentially expressed genes to known biological ontologies based on the GO project [37]. GO terms to which the different ORFs belong were obtained from the PATRIC database [27]. GO terms not present in the PATRIC database and whose annotation was obtained from the HOMD database or from the J. Craig Venter Institute were acquired using the program blast2GO under the default settings [38].

Enrichment analysis on these sets was performed using the R package GOseq, which accounts for biases due to over-detection of long and highly expressed transcripts [39]. Gene sets with ≤10 genes were excluded from analysis. We used the REVIGO web page [40] to summarize and remove redundant GO terms from the results. Only GO terms with FDR <0.05 were used. REVIGO plots were obtained for biological processes categories.

### Quantification of putative virulence factors

To identify putative virulence factors we used the Virulence Factors of Pathogenic Bacteria Database (VFDB) [41]. A similar approach, but with less stringent conditions, has been used by other authors to identify putative virulence factors in genomic islands [42]. The VFDB contains 1,205 virulence factors and 5,955 virulence factor related genes from 75 pathogenic bacterial genera [43]. We performed a BLAST similarity search of encoded proteins from the genomes in our database against the VFDB, with an e-value cutoff of 10 to 25 and identity >99% to exclude distant homologs.

### Integration of metatranscriptomic results with clinical traits

To integrate omics results with clinical parameters we used the R package mixOmics [44,45]. We calculated the sparse partial least square (sPLS) correlations between the clinical traits and species frequencies and profiles of gene expression in the progressing sites. Metatranscriptome hits were normalized by frequencies obtained in the metagenome before mixOmix analysis. For gene expression profiles, low count genes were filtered using the NOISeq function filtered.data with filter 1 and a minimum count per million of 30 [36]. Correlation Circle plots were obtained on the sPLS results to visualize associations between principal components species and gene expression profiles. Relevance networks showing correlations between genes and clinical traits were visualized in Cytoscape [46] with a threshold correlation of 0.95.

## Results

### Phylogenetic differences between sites in metagenome and metatranscriptome composition

**Figure 1:**
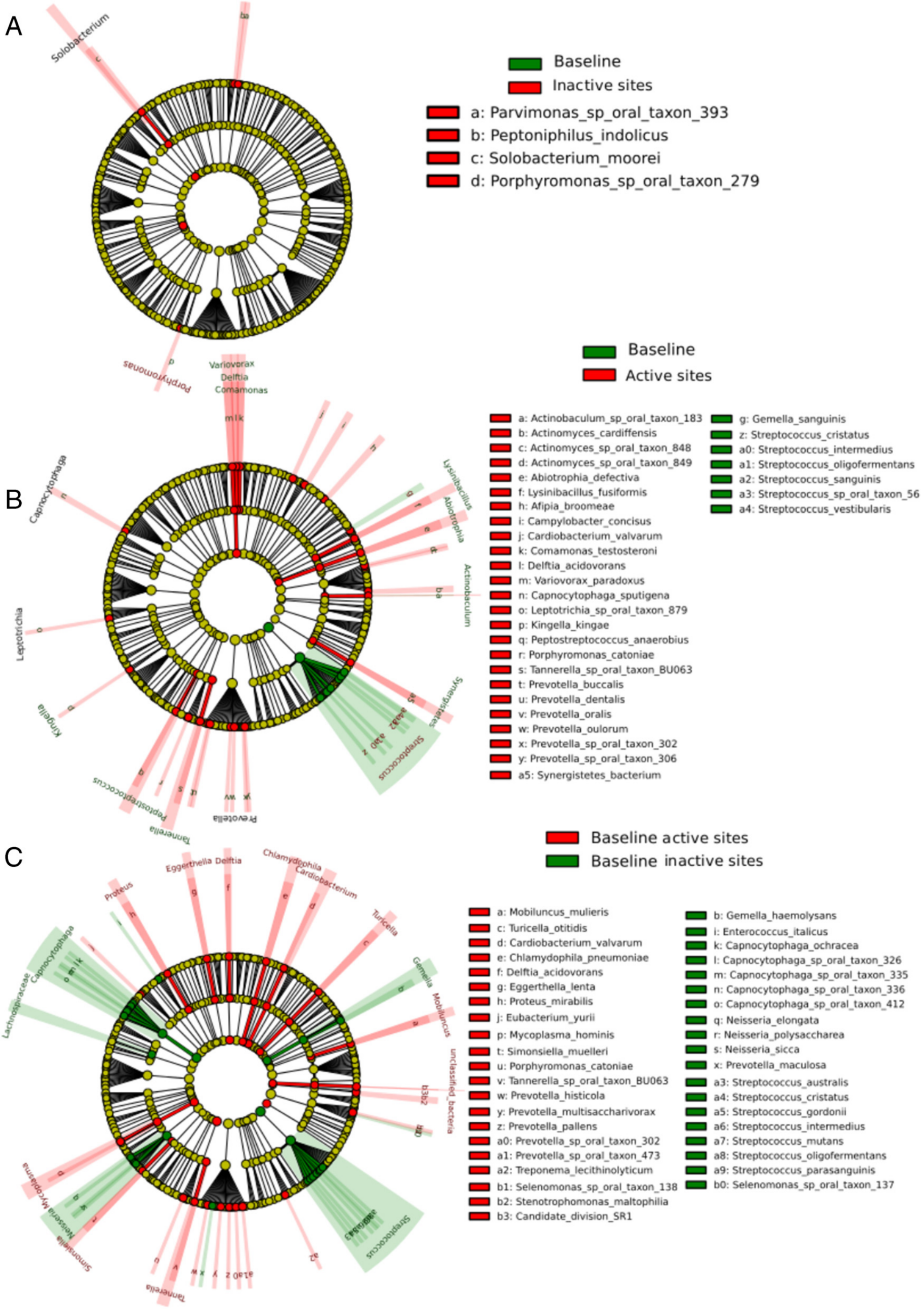
**Statistical differences in metagenome composition.** Metagenome hit counts were first normalized using GASIC. Normalized counts were then analyzed using LEfSe with default parameters, to identify significant differences at species level between the microbial communities compared. **(A)** Comparison baseline samples from active sites vs. progressing samples from active sites (that is, samples collect at the visit when an increase in CAL ≥2 mm was detected). **(B)** Comparison baseline samples from stable sites vs. follow-up samples from stable sites (that is, collected 2 months after baseline). **(C)** Comparison baseline samples from active sites vs. baseline samples from stable sites.

The comparison of phylogenetic assignments of the metagenome is presented in Figure 1. Two major observations can be derived from these results. First, changes in the metagenome of non-progressing sites were minor, only four species were significantly more abundant in the community at the endpoint of our study (Figure 1A). Second, differences in progressing sites were more significant (Figure 1B) and in those *Streptococcus* spp. dominated the community at baseline compared with the progressing community. What was more striking was the complete rearrangement at the metagenome level between the baselines of sites that did not progress versus sites that did progress (Figure 1C). Additionally, we compared the metagenome of baseline from progressing sites and non-progressing sites with samples from healthy sites of periodontally healthy individuals from a previous study [1]. The metagenome composition of both baseline communities is altered when compared to healthy communities (Additional file 2: Figure S1). *Streptococcus* spp. were more abundant in health than in the non-progressing baseline, while known periodontal pathogens such as *T. denticola* and *T. forsythia* were more abundant in the baseline samples (Additional file 2: Figure S1A). However, *Streptococcus* spp. were more abundant in the baseline from progressing sites than in healthy samples (Additional file 2: Figure S1B).

**Figure 2:**
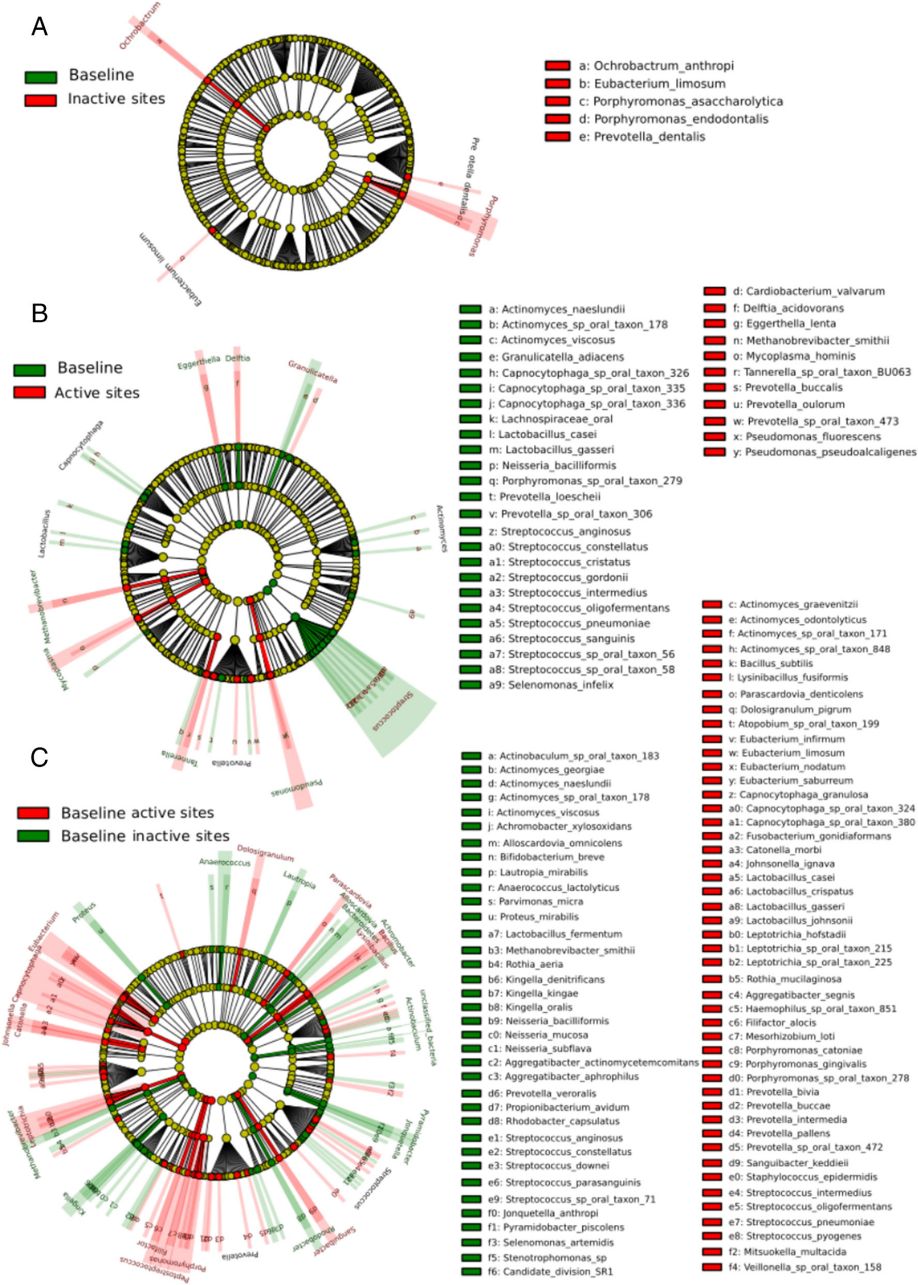
**Statistical differences in metatranscriptome normalized composition.** Metatranscriptome hits were first normalized by the relative frequency of species obtained in the metagenomic analysis using GASIC. Normalized counts were then analyzed using LEfSe with default parameters to identify significant differences in activity at the species level. **(A)** Comparison baseline samples from active sites vs. progressing samples from active sites (that is, samples collect at the visit when an increase in CAL ≥2 mm was detected). **(B)** Comparison baseline samples from stable sites vs. follow-up samples from stable sites (that is, collected 2 months after baseline). **(C)** Comparison baseline samples from active sites vs. baseline samples from stable sites.

We then looked at the fraction of the active community under the conditions studied. To perform these analyses we first normalized the metatranscriptome results by the relative abundance of the different species to obtain differences in expression due to real changes in levels of gene expression and not to increases in numbers of certain members of the community. Species frequencies were estimated using GASIC [33]. Figure 2 shows the results of these analyses. As in the case of the metagenome, only a few species were significantly more active in the non-progressing sites at the end of the study (Figure 2A). When we compared progressing sites to their baseline, and baselines from progressing and non-progressing sites, the differences were larger (Figure 2B and C). *Streptococcus* spp. dominated the activity of the community at baseline of progressing sites (Figure 2B). Moreover, a member of the red complex, *P. gingivalis*, several members of the orange complex including *P. intermedia* and *E. nodatum,* and the putative periodontopathogen *Filifactor alocis* were more active at the baseline of active sites than the baseline of non-progressing sites (Figure 2C).

As with the metagenome, we also compared the results of activity at baseline and time of progression with the activity of the community in healthy samples. When comparing baseline of non-progressing sites to healthy sites, the first obvious results was that a larger fraction of the community is more active in the baseline samples than in health, even though they were clinically similar (Additional file 2: Figure S2). The same could be said for the baseline samples of sites that progressed; a large fraction of the community was significantly more active than the subgingival communities in health (Additional file 2: Figure S3).

### Community-wide changes in patterns of gene expression in non-progressing and progressing sites during periodontitis progression

**Figure 3:**
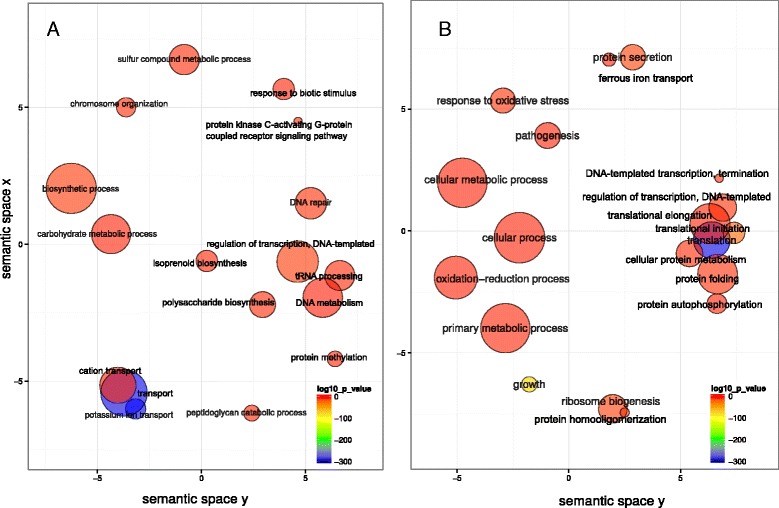
**GO enrichment analysis comparing baseline in active sites to progression profiles in the same sites.** Enriched terms obtained using GOseq were summarized and visualized as a scatter plot using REVIGO. **(A)** Summarized GO terms related to biological processes at baseline. **(B)** Summarized GO terms related to biological processes in progression. Circle size is proportional to the frequency of the GO term, while color indicates the log10 *P* value (red higher, blue lower).

We first characterized differences in gene expression between baseline and progression in sites that showed disease activity during monitoring. We analyzed the global behavior of the community by identifying enrichment of GO terms. These results represent global changes in the community and could be due to over-expression of certain genes as well as increase in members of the community whose contribution to the observed activities is now higher due to their larger number. Potassium and amino-acid transport, peptidoglycan catabolism, isoprenoid biosynthesis, polysaccharide biosynthesis, and protein kinase C-activating G-protein coupled receptor signaling pathway were over-represented activities at baseline when compared to the end point of progression (Figure 3A). Over-represented activities at sampling time in progressing sites are shown in Figure 3B. We observed an over-representation of pathogenesis associated GO terms as well as activities related to response to oxidative stress.

We normalized the hit counts against species frequencies estimated using GASIC [33]. These normalized counts represented actual over-expression at the species level and not just increase in the total number of individuals in the community. Interestingly, the list of differentially expressed (DE) genes before and after normalization was very similar (Additional file 2: Figure S4). A total of 132,251 of the DE genes were identical in both, normalized and no-normalized gene sets; 412 DE genes were only identified exclusively in the normalized list and only 6,126 in the no-normalized set of DE genes. These results suggest that most of the differences observed were due to changes in gene expression at the species level rather than changes in the numbers of the members of the community.

When we compared the expression profiles of baseline and follow-up samples from non-progressing sites (that is, sites that did not change based on CALs) we did not identify any gene as differentially expressed, indicating that clinically stable sites did not have significant changes in gene expression during the period we studied those sites.

Changes in gene expression profiles in major periodontal pathogens members of the red complex (*P. gingivalis*, *T. denticola,* and *T. forsythia*) during periodontal disease progression showed that upregulated genes belonged to GO terms associated with transport (iron, cation, lactate, citrate, sodium, and phosphate), proteolysis, protein kinase C-activating G-protein coupled receptor signaling pathway and response to antibiotic (Additional file 2: Figure S5A), while downregulated genes belonged to GO terms associated with cobalamin (vitamin B12) biosynthesis (Additional file 2: Figure S5B). Individually we observed that *Treponema denticola* upregulated genes related to flagella biosynthesis (*fla*A, *fla*G, *fli*Q, and *fli*W), oligopeptide ABC transporters, and a large number of hypothetical proteins (Additional file 3: Table S1). *Tannerella forsythia* and *Porphyromonas gingivalis* both upregulated different TonB-dependent receptors, genes involved in iron transport (ferric uptake siderophores and ferrous iron transport protein B), a large number of peptidases and proteases including ClpB, genes associated with aerotolerance (*Bacteroides* aerotolerance operon *bat*A-E and *mox*R-like ATPase of the aerotolerance operon), and CRISPR- associated genes (*csp*, *csm,* and *cas* genes) (Additional file 3: Table S1).

Finally, *P. gingivalis* specifically upregulated genes related to biotin synthesis (biotin synthase, bioC, and bioG), capsular polysaccharide biosynthesis proteins and a large number of proteins of conjugative transposons (*tra*A, *tra*B, *tra*E, *tra*F, *tra*G, *tra*I, *tra*J, *tra*K, *tra*L, *tra*M, *tra*N, *tra*O, *trap*, and *tra*Q) and transposases (ISPg2, ISPg3, ISPg4, ISPg5, and ISPg6). *T. forsythia* specifically upregulated transposases (IS116, IS110, IS902, and IS4 families) and large numbers of different homologs of SusC and SusD family proteins, involved in polysaccharide binding. Regarding downregulated proteins of the red complex most of them were hypothetical in all three of its members (Additional file 4: Table S2).

Profiles of expression of the members of the orange complex were very similar to the ones from the red complex. They upregulated different TonB-dependent receptors, a large number of peptidases and proteases including ClpB, genes associated with aerotolerance (*Bacteroides* aerotolerance operon *bat*A-E i and *mox*R-like ATPase of the aerotolerance operon in *P. intermedia* and *P. nigrescens*), genes involved in iron transport (ferric uptake siderophores and ferrous iron transport protein B), hemolysins, CRISPR- associated genes (in *C. gracilis*, *C. rectus*, *C. showae*, *P. nigrescens,* and *S. constellatus*) and chaperones GroEL, GroES, and GrpE (Additional file 5: Table S3). As in the case of *P. gingivalis*, both *P. intermedia* and *P. nigrescens* upregulated a large number of mobilization genes from conjugative transposons (*tra*A, *tra*B, *tra*D, *tra*E, *tra*F, *tra*G, *tra*I, *tra*J, *tra*K, *tra*L *tra*M, *tra*N, *tra*O, and *tra*Q) (Additional file 5: Table S3).

### Comparison of metatranscriptomic profiles from progressing vs. non-progressing sites at baseline

**Figure 4:**
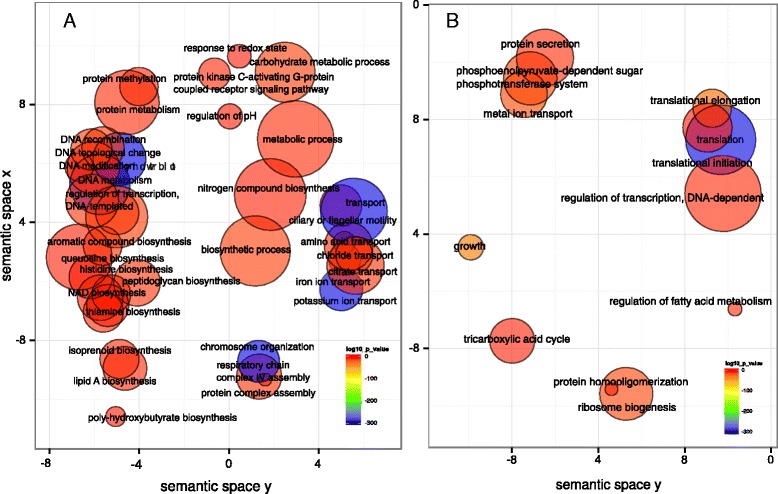
**GO enrichment analysis comparison of baselines from progressing and non-progressing sites.** Enriched terms obtained using goseq were summarized and visualized as a scatter plot using REVIGO. **(A)** Summarized GO terms related to biological processes in baselines of progressing sites. **(B)** Summarized GO terms related to biological processes in baselines of non-progressing sites. Circle size is proportional to the frequency of the GO term, while color indicates the log10 *P* value (red higher, blue lower).

In order to identify activities that could be related to the initial steps of disease progression we compared community-wide expression profiles of samples at baseline from sites that did not progress vs. sites that did. The analysis of GO enrichment terms showed an over-representation in the baseline of progressing sites of terms related to cell motility, transport (iron, potassium, chloride, citrate, and amino acids transport), lipid A and peptidoglycan biosynthesis, protein kinase C-activating G-protein coupled receptor signaling pathway, as well as synthesis of aromatic compounds (Figure 4A). On the other hand, in the baseline samples from non-progressing sites there was an over-representation of GO terms related to tricarboxylic acid cycle, metal ion transport, phosphoenolpyruvate-dependent sugar phosphotransferase system and protein secretion (Figure 4B).

When compared baselines with healthy sites we found that the clinically healthy sites at baseline from diseased individuals were already impacted by disease. Both baselines from progressing and non-progressing sites had an over-representation of GO terms associated with citrate, organic ion, and lactate transport, as well as sulfur compound metabolic processes and peptidoglycan catabolism (Additional file 2: Figure S6).

Differences in gene expression of the red complex between baseline samples from progressing vs. non-progressing showed similar enrichment of activities than when studying progressing sites. GO assignment of the differentially expressed genes showed association with proteolysis, sulfur compound metabolism and response to antibiotic in upregulated genes and translation in downregulated genes (Additional file 2: Figure S7). *P. gingivalis* actively upregulated putative virulence factors (34 in total) while *T. denticola,* with three upregulated putative virulence factors, and *T. forsythia,* with one, seem not especially active at this stage (Additional file 6: Table S4).

A more complex picture emerged when we analyzed the behavior of the orange complex. As a whole the members of the orange complex (*P. intermedia, P. nigrescens, P. micra, F. nucleatum, F. periodonticum, C. gracilis, C. rectus, S. constellatus, E. nodatum,* and *C. showae*) showed upregulation of genes associated with proteolysis, sodium ion transport, cellular response to phosphate starvation, and regulation of pH (Additional file 2: Figure S8A).

### Expression of putative virulence factors in the oral community during periodontitis progression and at baseline of progressing vs. baseline of non-progressing sites

**Figure 5:**
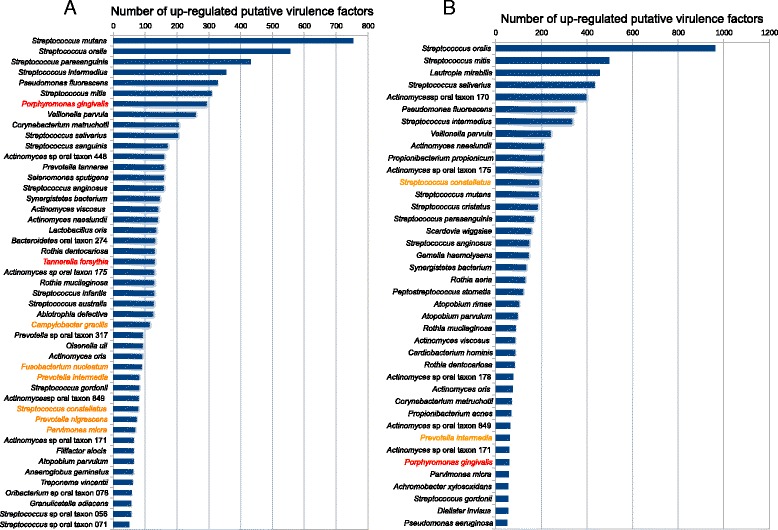
**Ranked species by the number of upregulated putative virulence factors in the metatranscriptome.** Putative virulence factors were identified by alignment of the protein sequences from the different genomes against the Virulence Factors Database (VFDB) as described in the Methods section. Numbers in the graph refer to the absolute number of hits for the different species for the putative virulence factors identified. The numbers correspond to the total hits to different strains corresponding to the species in our database and they could be redundant. In red are members of the red complex. In orange are members of the orange complex. **(A)** Comparison of baseline to progressing. **(B)** Comparison of baseline non-progressing to baseline progressing.

In the case of progressing sites, when comparing baseline vs. break down visit, a total of 9,147 hits of putative virulence factors from 207 species were identified in the genes over-represented in progressing samples. Nonetheless, not all of them expressed a large number of them, only 47 showed upregulation of 50 or more putative virulence factors under these conditions. Two members of the red complex, *P. gingivalis* and *T. forsythia,* expressed a large number of the putative virulence factors in the progressing samples (Figure 5A). More active in these samples were members of the orange complex: *C. gracilis, F. nucleatum, P. intermedia, S. constellatus, P. nigrescens,* and *P. micra* (Figure 5A). Three members of this complex were especially active in the upregulation of putative virulence factors: *C. gracilis, F. nucleatum,* and *P. intermedia* upregulated 114, 90, and 82 genes, respectively, that have homology with virulence factors in our database (Additional file 7: Table S5). *P. micra* upregulated 69 putative virulence factors, *P. nigrescens* 73*, S. constellatus* 79*, C. rectus* 13, and *C. showae* 11. We did not identify any putative virulence factor upregulated from *E. nodatum* or from *F. periodonticum.* When we looked at the global activities associated with the expression of these virulence factors we obviously observed an over-representation of GO terms related to pathogenesis as well as iron transport and lipid A biosynthesis (Additional file 2: Figure S9A). Focusing on the members of the red complex we could also see as common themes an up-regulation of genes associated with iron transport and lipid A biosynthesis (Additional file 2: Figure S9B). More interestingly were the results associated with the orange complex. In addition to the same activities mentioned for the red complex, members of the orange complex upregulated genes involved in cell adhesion, proteolysis, and pilus assembly during progression (Additional file 2: Figure S9C).

We then looked at the global activities associated with the expression of virulence factors comparing baselines of non-progressing vs. progressing sites and found that in the baseline of progressing sites the major activities corresponded to pathogenesis and ferrous ion transport while in non-progressing baselines there was an over-representation of GO terms related to cobalamin biosynthesis and sodium ion transport (Additional file 2: Figure S10). Comparing the expression of virulence factors at baseline gave a better understanding of the role of two complexes (red and orange) in the first stages of disease. We did not observe a significant over-representation of any GO terms in any of the members of the red complex. Nonetheless, members of the orange complex were indeed upregulating genes involved in proteolysis and iron homeostasis (Additional file 2: Figure S11). Surprisingly enough, we identified a set of organisms that were highly active, transcribing genes of putative virulence factors that have not been usually associated with periodontal disease. *S. oralis*, *S. mutans*, *S. intermedius*, *S. mitis*, *V. parvula,* and *P. fluorenscens* were upregulating a large number of putative virulence factors in both analysis (baseline vs. progression, comparing baseline in progression with baseline in non-progressing sites) (Figure 5).

All of the above cited organisms upregulated different oligopeptide transport systems (*opp*A, *opp*D, *opp*F, and *opp*B). *S. oralis*, *S. mutans*, *S. intermedius*, *S. mitis, P. fluorenscens,* and *V. parvula* upregulated several hemolysins, manganese ABC transporters, manganese superoxide dismutase, and a protein serine threonine phosphatase (PrpC) involved in regulation of stationary phase (Additional file 8: Table S6). *S. oralis, S. mitis,* and *V. parvula* upregulated vitamin B12 ABC transporters (Additional file 8: Table S6). *S. oralis*, *S. mutans*, *S. intermedius,* and *S. mitis* all upregulated Clp protease and LytR trancriptional attenuator. *P. fluorescence* upregulated all genes associated with flagellar synthesis (*fla*A*, fla*G*, fle*N*, flg*A*, flg*C*, flg*F*, flg*I*, flg*J*, flg*K*, flg*N, *flh*A, *flh*B, *flh*F, *fli*D, *fli*F, *fli*G, *fli*I, *fli*K, *fli*N, *fli*P, *fli*Q, *fli*R, *fli*S, and *mot*B) and genes related to chemotaxis (Additional file 8: Table S6).

In spite of the commonalities in upregulated genes when comparing baselines of progressing to non-progressing sites and progression, there were also specific signatures of the two comparisons. For instance, *S. oralis* and *S. mitis* upregulated collagen adhesion proteins and *V. parvula* TonB-dependent receptors when comparing baselines but not during progression.

### Integrating expression profiles and clinical traits during periodontitis progression

Integrating microbiological functions with clinical parameters is still one of the challenges in omics analysis. We used multivariate statistical analysis and visualization tools implemented in the R package mixOmics [44] to identify relevant associations between gene expression and certain clinical traits: bleeding on probing (BOP), increase in pocket depth (ΔPD) and increase in clinical attachment level (ΔCAL). We calculated the sparse partial least square (sPLS) correlations between the evolution of clinical traits and profiles of gene expression in the progressing sites. Additional file 2: Figure S12 visualizes of the relationships using correlation circle plots. Not surprisingly, ΔPD and ΔCAL were highly correlated between them (Additional file 2: Figure S12A). There was a set of genes whose expression profiles were highly correlated with ΔPD and ΔCAL evolution (Additional file 2: Figure S12A). There were no genes whose profiles correlated with BOP (Additional file 2: Figure S12). Interestingly, two large set of genes correlated with other components (Additional file 2: Figure S12B), but which possibly corresponded to another clinical trait not analyzed in our study.

**Figure 6:**
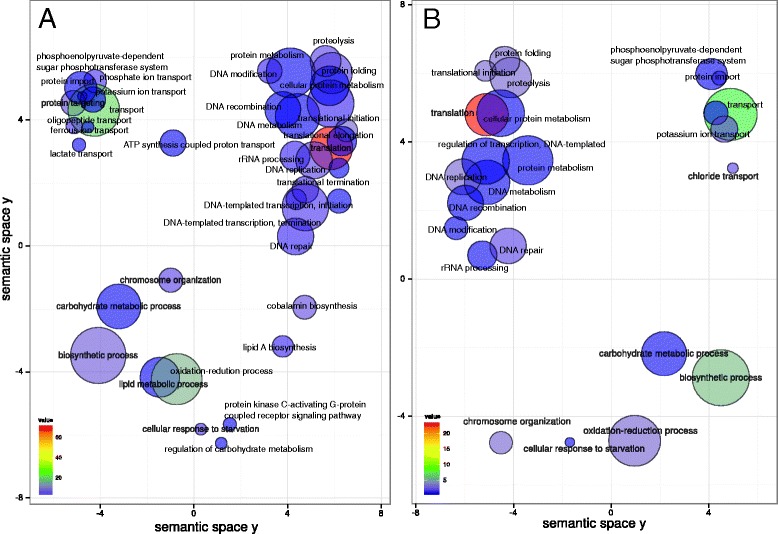
**GO assignment terms for genes with relevance networks associations with clinical parameters.** Relevance networks were obtained using the first three sPLS dimensions. We used a threshold of r = 0.95 to select for association between progression of clinical parameters (ΔPD increase in pocket depth, ΔCAL increase in clinical attachment level) and gene expression profiles. GO terms were assigned to genes whose pattern of expression was significantly associated with the clinical parameters measured. GO terms were summarized using REVIGO. **(A)** GO terms associated with ΔPD. **(B)** GO terms associated with ΔCAL.

We also analyzed correlation structures between clinical traits and gene expression using relevance networks [44]. This method generates a graph where nodes represent variables and the edges represent the correlations. The correlation of gene expression profiles gave a large number of genes that correlated with the clinical parameter profiles. Not surprisingly, BOP, which is a dichotomous variable with only two states (yes or no), did not correlate with any gene profiles. ΔPD and ΔCAL correlated with a large number of gene profiles, even with an r = 0.95. We then assigned GO terms to the correlated genes and summarized these results using REVIGO. We detected specific patterns of activities associated with increases in PD and CAL (Figure 6A and B). Among those patterns we found that the profiles of expression of phosphoenolpyruvate-dependent sugar phosphotransferase system, proteolysis and potassium transport were associated with the worsening of those two clinical parameters.

### Viral activity in the oral cavity during progression

We also looked at the presence of eukaryotic viruses and bacteriophages, which have been previously associated with disease [47] and may play a role in shaping the bacterial community [48]. We identified viral activities in all samples although the number of transcripts represented a small fraction of all hits identified, between 0.04% and 0.7% of all hits were of viral origin (Additional file 2: Figure S13). To confirm that those hits really belong to viral sequences we obtained consensus sequences from the .bam files of our alignments and BLASTed them against the nr database at NCBI. Consensus sequence from 14 out of the 16 samples analyzed had sequences with significant matches to viral sequences. When we compared the relative activities of viruses at baseline and progression, we observed high activity of phages and herpesvirus in the progressing sites in relation with the baseline samples (Additional file 2: Figure S14).

## Discussion

In a recent report we used a combined metagenomic/metatranscriptomic approach to characterize the functional dysbiotic phenotype of the oral microbiome during severe chronic periodontitis. We took advantage of next generation sequencing (NGS) techniques and the fact that a large number of genomes from oral isolates have been sequenced [49] to infer functional differences between the subgingival microbiota of periodontal health and chronic severe periodontitis [1]. To date, only a few studies have been used to study the transcriptome of the community-wide oral microbiome, either to characterize microbial expression in caries [50,51] or periodontitis [52] but none of them addressed the question of why some dental site suffer peridontitis progression while other remain stable.

Recent studies on community composition that used NGS analysis compared samples from periodontally healthy subjects with chronic periodontitis samples reached similar conclusions and suggested that members of the genera *Prevotella*, *Fusobacterium*, *Treponema*, *Sinergistetes, Filifactor,* and *Porphyromonas* and candidate division TM7 were more abundant in periodontitis whereas *Actinomyces* and *Streptococcus* were less frequent in samples from periodontitis compared to periodontally healthy subjects [10,11]. These results agreed with the associations of red and orange complexes with periodontal disease previously postulated using other detection methods [9,53]. We observed similar results in our previous study when comparing health and severe chronic periodontitis [1]. Although most analyses were performed using 16S rDNA sequencing and our current study used metagenomic analysis (shot gun sequencing using NGS), we also found that in the progressing sites *Prevotella* and *Sinergistetes* were more abundant when the teeth broke down than at baseline while the opposite was true for the genus *Streptococcus*. Non-progressing sites had almost the same metagenomic composition at baseline and at the 2-month visit, when another sample was taken.

Examining baseline samples might give us insights into the changes in microbial composition and activities that define the initial stages of progression. Large differences were observed when comparing baseline samples of progressing with non-progressing sites. Members of the genera *Porphyromonas*, *Treponema, Tannerella,* and *Prevotella*, among others, were more abundant at baseline on sites that progressed. Members of the genus *Streptococcus* were more abundant at baseline in non-progressing sites.

We also looked at differences in the phylogenetic assignment of active members of the microbial community. We found that, as in the metagenome, no major differences were observed in stable sites when comparing baseline and 2-month visit samples. However, the differences where profound when we compared progressing sites at baseline with the breakdown time point and even more profound when we compared the two baseline samples from progressing and non-progressing sites. During progression *Streptococcus* spp. and *Actinomyces* spp. were highly active at baseline. In contrast *Mycoplasma* spp. were more active in progression. Although little attention has been paid to *Mycoplasma* spp. as potentially important in oral diseases in most studies, it has been significantly associated with periodontitis [10,11,54,55]. Interestingly, *Synergistites* sp. and the archaea *Methanobrevibacter* sp. were more active during progression. These microorganisms have been previously associated with periodontal disease [10,11,56].

When we looked at differences at baseline between active and non-progressing sites we found that the known periodontal pathogens *T. denticola* and *T. forsythia* were not significantly more active while *P. gingivalis* was. *P. gingivalis* has been proposed as a keystone-pathogen in periodontitis. The keystone-pathogen hypothesis states that specific low-abundance microbes can lead the process of periodontal inflammation and tissue destruction by transforming a normally healthy microbiome into a dysbiotic state [57,58]. The high activity of *P. gingivalis* at baseline of active sites will agree with this hypothesis and it would indicate that even at early stages *P. gingivalis* is starting to be involved in the dysbiotic process that leads to progression.

Additionally, *F. alocis*, *E. nodatum,* and several *Prevotella* spp. were more active in the sites that would progress than in the site that would remain non-progressing. Both *F. alocis* and *E. nodatum*, a member of the orange complex, have been previously associated with periodontal disease [55,59-61].

Testing for differential representation of GO terms gives an overall view of the metabolic activities of the whole community under different environmental conditions. Interestingly, when we compared the expression profiles of longitudinal samples from stable sites, we did not identify any gene as differentially expressed. This indicates that the community as a whole did not change its expression patterns in the two months between the first visit and the time when subsequent samples were taken.

When we looked at progression, the results pointed to several functional signatures characteristic of the active sites. At the breakdown point active sites were actively expressing genes associated with pathogenesis, response to oxidative stress and ferrous iron transport. Oxidative stress is one of the consequences of the host inflammatory response to the microbial challenge [62] and bacteria must act to defense themselves against this host defense mechanism, which is probably accentuated with the progression of disease. Iron is an essential enzymatic cofactor and we have already shown the *in situ* over-expression of genes related to its transport in the microbial community during severe chronic periodontitis [1]. At baseline, GO terms associated with isoprenoid and polysaccharide biosynthesis, sulfur compound metabolic processes, potassium ion transport and protein kinase C-activating G-protein coupled receptor signaling pathway were highly enriched. Lipopolysaccharide (LPS) is a key factor in the development of periodontitis [63,64] and high levels of lipopolysaccharide (LPS) from *P. gingivalis* have been reported to delay neutrophil apoptosis and provide a mechanism to modulate the restoration and maintenance of inflammation in periodontal tissues [65-67]. Hydrogen sulfide production from aminoacids and peptides has been reported in periodontal bacteria and the different efficiency of use of these compounds could be important determinants of the periodontal microbial ecology [68,69]. More puzzling is the over-representation of GO terms related to potassium transport. Potassium transport systems have been associated with pathogenesis in other organisms such as *Staphylococcus aureus* [70] and *Salmonella* [71], but not in oral bacteria. Interestingly, significant higher levels of potassium have been reported in periodontitis in both gingival crevicular fluid (GCF) and saliva [72,73].

Comparing baseline metabolic activities of non-progressing vs. progressing sites might give us a better understanding of the initial stages of disease and the role that the microbial community plays at this early stage of pathogenesis. Among those functional signatures we found: citrate transport, iron transport, potassium transport, amino-acid transport, isoprenoid biosynthesis, and ciliary and flagellar motility. Citrate transport has been linked to iron transport and virulence in other organisms, such as *Shigella* and *Pseudomonas* [74,75]. This is in accordance with our previous observations in severe chronic periodontitis sites [1]. As mentioned above, the efficiency in utilizing various amino acids and peptides is among the key determinants of the periodontal microbial ecology [68] and its uptake may give additional advantages to certain members of the microbial community. In the active sites, there seems to be a shift from amino-acid uptake to oligopeptide uptake throughout the breakdown process. Isoprenoid biosynthesis, probably involved in the synthesis of peptidoglycan, was also over-represented in active sites. Isoprenoids are a large, diverse class of naturally occurring organic chemicals which are essential for cell survival. The 2C-methyl-D-erythritol 4-phosphate (MEP) pathway has been implicated in the virulence of *Listeria monocytogenes*, *Mycobacterium tuberculosis, and Brucella abortus* [76], and evidence suggests that the MEP pathway may be involved in intracellular survival by combating oxidative stress [77]. Moreover, a metagenomic analysis of the human distal gut microbiome revealed that MEP pathway genes are highly abundant in that community; perhaps reflecting the abundance of the MEP pathway in bacteria in general [78]. Finally, ciliary and flagellar motility as well as chemotaxis genes that could direct bacterial movement were all part of the signature activities at the initial stages of progression. Motile pathogenic members of the oral community, such as *Treponema* spp., possess the capacity for tissue invasion thanks to the synthesis of flagella [79,80]; our results suggest that this fraction of the community is already active at the initial stages of progression.

Historically, members of the red and orange complexes have been associated with chronic periodontitis [7,9]. Consistent with their postulated role in progressing sites, we observed a high level of expression of putative virulence factors by members of both complexes when breakdown was clinically detected. However, at the baseline of our studies it seems that the relative importance of these complexes in the active sites is reduced. Only *P. gingivalis*, *S. constellatus,* and *P. intermedia* were actively expressing putative virulence factors.

Interestingly, members of the red complex showed enrichment in response to antibiotics (beta-lactamase activity) during progression and even at baseline of progressing sites. We observed the same phenomenon at whole community-level in our previous study comparing healthy sites vs. chronic severe periodontitis [1]. Beta-lactamase activity has been observed in adult periodontitis at low-level enzymatic activity but with high prevalence [81] and seems to be a frequent phenomenon in samples from polymicrobial diseases [82]. We still do not know what role this enzymatic activity plays on the progression of the disease given that the patients of this study were not treated with antibiotics at the time of sampling.

CRISPR- associated genes in *P. gingivalis*, *T. forsythia*, *C. gracilis*, *C. rectus*, *C. showae*, *P. nigrescens,* and *S. constellatus* were highly upregulated during progression. We observed phage activity in all samples analyzed, which could explain the high level of production of CRISPR-associated proteins as a mechanism of defense against viral activity [83]. However, we cannot discard the possibility that CRISPR-associated proteins are playing a broader role in the virulence mechanisms of periodontitis. Thus, recently CRISPR-Cas systems have been linked to stress responses and virulence in bacteria [84] and to competitive interactions between members of the red complex [85].

*P. gingivalis*, *P. nigrescens,* and *P. intermedia* upregulated all the *tra*A-Q and *mob* genes in their chromosomal conjugative transposons. These genes are required for formation of a conjugal pore and DNA mobilization [86,87]. The upregulation of these genes could indicate conjugative transposons mobilization in *Porphyromonas* and *Prevotella* which would agree with evidence of natural horizontal transfer of antibiotic resistance through conjugative transposon mobilization in those organisms [87-89]. However, the mobilization of those conjugative transposons was most likely driven by the presence of antibiotics. In the current study, subjects did not use systemic antibiotics during the monitoring period when samples were collected. Therefore, it is not clear what signal(s) triggered this mobilization of conjugative transposons. It is noteworthy that we did not observe this phenomenon in severe chronic periodontitis samples [1].

The idea that the whole community acts as a pathogen rather than only specific organisms has been gaining traction in recent years [1,90,91]. In agreement with this hypothesis we found that a group of organism not usually considered pathogens were upregulating a large number of putative virulence factors in active sites. Among these groups, we observed that some streptococci, including *S. mitis* and *S. intermedius,* were especially active. Although *S. mitis* and *S. intermedius* are usually associated with periodontal health, they have also been found to form part of the community in periodontitis [10,11]. We found *V. parvula* highly active in both progressing and baseline sites, which was surprising since *V. parvula* is almost always associated with a healthy community [10,23]. However, streptococci and *V. parvula* have been identified as part of a cluster associated with periodontitis in adolescents [92]. Another surprising finding was the identification of *P. fluorescens* as one of the top producers of virulence factors. This is not an organism usually associated with periodontitis, although another member of its genus, *P. aeruginosa*, has indeed been associated with other important pathologies such as cystic fibrosis [93,94]. In our previous study on chronic severe periodontitis, we also observed a similar behavior where the whole community, and not only known periodontal pathogens, expressed more putative virulence factors in diseased sites. Among the most active producers of putative virulence factors was *Corynebacterium matruchotii* [1], which has also been associated with periodontitis in microbiome studies. Interestingly, we also found this organism as highly active in progressing sites but not at the baseline, indicating a shift into a pathogenic microbial community.

We then established association between profiles of clinical parameters such as BOP, ΔPD, and ΔCAL with profiles gene expression. BOP showed no association with changes in gene expression profiles. This is not surprising since BOP is a discrete variable. Nonetheless, ΔPD and ΔCAL were highly associated with proteolytic activity and potassium ion transport. Additionally, ΔPD was associated with cobalamin biosynthesis and ferrous and oligopeptide transport. Proteolysis has been recognized as an important virulence determinant in periodontitis progression [95,96]. In the case of vitamin B12 (cobalamin) synthesis, we observed an upregulation of the vitamin B12 ABC transporter *btu*FCD system in *P. gingivalis* and *T. forsythia* and some members of the orange complex*. P. gingivalis* harbors all the genes necessary to convert precorrin-2 into cobalamin, but it lacks the genes for the synthesis of precorrin-2 [97]. In our previous study on chronic severe periodontitis we also observed an up-regulation of *btu*FCD system in *P. gingivalis* and *T. forsythia* [1]. An increase in synthesis and release to the external medium by other organisms of cobalamin might give members of the red and orange complex an ecological advantage if they start scavenging it.

## Conclusions

The initial causes for transition from a healthy microbial community to a dysbiotic one are still not well understood in great part due to the complexity of the oral community. Using a metagenomic/metatranscriptomic approach, and comparing baseline samples from the same individuals, we have begun the study of the physiological changes in the microbial community that are associated with the initial stages of dysbiosis. Here, we show that in periodontitis progression there are certain characteristic activities that are associated with the onset of breakdown in specific teeth. Among those we found that citrate transport, iron transport, potassium transport, amino-acid transport, isoprenoid biosynthesis, and ciliary and flagellar motility are signatures of the initiation of periodontitis progression.

The data presented here illustrate that regardless of the overall composition of the community, certain metabolic signatures are consistent with disease and progression. For instance, the community composition in the progressing active sites was relatively different from the Massachusetts in chronic severe periodontitis sites previously described [1]. Nonetheless, in both cases we observed that iron transport and protein secretion activities are highly associated with advance stages of disease. Moreover, as we have shown in our previous study on chronic severe periodontitis [1], our results show that the whole community, and not just a handful of oral pathogens, is responsible for an increase in virulence that could lead to progression. Finally, we found that certain ecological changes could explain the evolution of certain clinical parameters. As we discuss above, an increase in production of cobalamin could exacerbate the growth of periodontal pathogens that lack the capabilities to synthesis this compound and explain, at least in part, the association of these organisms with increase in disease severity.

### Availability of supporting data

The datasets used in these analyses were deposited at the Human Oral Microbiome Database (HOMD) under the submission numbers 20130522 [98] and 20141024 [99].

## Additional files

Additional file 1: Table S7. List of genomes used in the study. We generated a database of genomes that contains 524 genomes from 312 species of Bacteria and one species of Archaea and was used for alignment of the sequences in our results. Additional file 2: Figure S1. Statistical differences in metagenome composition. **Figure S2.** Statistical differences in normalized metatranscriptome composition comparing non-progressing sites baselines to healthy sites of healthy patients. **Figure S3.** Statistical differences in normalized metatranscriptome composition comparing progressing sites baselines to healthy sites of healthy patients. **Figure S4.** Venn diagram showing overlapping differentially expressed genes with and without normalization. **Figure S5.** GO terms associated with changes in gene expression profiles in major periodontal pathogens members of the red complex during periodontal disease progression. **Figure S6.** GO enrichment analysis comparing healthy sites from healthy individuals and baselines in progressing sites and non-progressing sites. **Figure S7.** GO terms associated with changes in gene expression profiles in major periodontal pathogens members of the red complex when comparing baselines of progressing and non-progressing sites. **Figure S8.** GO terms associated with changes in gene expression profiles in members of the orange complex when comparing baselines of progressing and non-progressing sites. **Figure S9.** GO terms associated with changes in gene expression of putative virulence factors in the oral community during periodontitis progression. **Figure S10.** GO terms enrichment analysis of virulence factors comparing baselines. **Figure S11.** GO terms enrichment analysis of virulence factors in the orange complex comparing baselines. **Figure S12.** Correlation circle plots of sPLS analysis. **Figure S13.** Percentage of hits corresponding to viral sequences. **Figure S14.** Statistical differences in viral composition of transcript libraries. Additional file 3: Table S1. Upregulated genes in members of the red complex during periodontal disease progression. After identifying upregulated genes of the whole community we selected those upregulated genes corresponding to the red complex (*Porphyromonas gingivalis, Treponema denticola,* and *Tannerella forsythia*), which are consider major periodontal pathogens. Additional file 4: Table S2. Downregulated genes in members of the red complex during periodontal disease progression. After identifying downregulated genes of the whole community we selected those downregulated genes corresponding to the red complex (*Porphyromonas gingivalis, Treponema denticola,* and *Tannerella forsythia*), which are consider major periodontal pathogens. Additional file 5: Table S3. Differentially expressed genes in members of the orange complex. Members of the orange complex (*P. intermedia, P. nigrescens, P. micra, F. nucleatum, F. periodonticum, C. gracilis, C. rectus, S. constellatus, E. nodatum,* and *C. showae*) have been also associated with chronic periodontitis. Here were showed DE genes of the members of this complex during periodontal disease progression. Additional file 6: Table S4. Differentially expressed (DE) putative virulence factors of the red complex when comparing the baseline expression profiles of active and inactive sites. Based on the VFDB (see Methods) we compared expression of DE putative virulence factors at baseline and selected those corresponding to the red complex. Additional file 7: Table S5. Differentially expressed (DE) putative virulence factors of the orange complex when comparing the baseline expression profiles of active and inactive sites. Based on the VFDB (see Methods) we compared expression of DE putative virulence factors at baseline and selected those corresponding to the orange complex. Additional file 8: Table S6. Upregulated virulence factors in highly active members of the community in progressing sites. Other members of the community, usually not considered pathogens, showed a large number of putative virulence factors being upregulated during periodontitis progression and at baseline of progressing sites. Here we summarized the results showing the species whose upregulation of putative virulence factors was high, along with locus ID and annotation of the genes according to PATRIC (See Methods).
