# Erratum to: Functional signatures of oral dysbiosis during periodontitis progression revealed by microbial metatranscriptome analysis

**DOI:** 10.1186/s13073-015-0231-6

**Published:** 2015-10-27

**Authors:** Susan Yost, Ana E Duran-Pinedo, Ricardo Teles, Keerthana Krishnan, Jorge Frias-Lopez

**Affiliations:** Forsyth Institute, 245 First Street, Cambridge, 02142 Massachusetts USA; Harvard School of Dental Medicine, 188 Longwood Ave, Boston, 02115 MA USA; University of North Carolina Chapel Hill, School of Dentistry, Chapel Hill, 27599-7450 NC USA

## Erratum

It has come to our attention that there is an error in the methods section of this article [1]. The sentence: “Quantified libraries were pooled and sequenced using the MiSeq v2, 2 × 150 cycle cartridge (Illumina)” is incorrect and should read: “Quantified libraries were pooled and sequenced using the MiSeq v3, 2 × 75 cycle cartridge (Illumina)”. The publisher apologises for any inconvenience caused.
