# Supplementary material for: Functional signatures of oral dysbiosis during periodontitis progression revealed by microbial metatranscriptome analysis
Source: Genome Med. 2015 Apr 27;7(1):27. doi: 10.1186/s13073-015-0153-3 (PMC4410737; doi:10.1186/s13073-015-0153-3)
Supplement: Additional file 1: Table S7. — List of genomes used in the study. We generated a database of genomes that contains 524 genomes from 312 species of Bacteria and one species of Archaea and was used for alignment of the sequences in our results. [file 13073_2015_153_MOESM1_ESM.doc]

| **Table S7. List of genomes used in the study.** We generated a database of genomes that contains 524 genomes from 312 species of Bacteria and 1 species of Archaea and was used for alignment of the sequences in our results. | |
| --- | --- |
| Abiotrophia defectiva ATCC 49176 | Mobiluncus mulieris ATCC 35239 |
| Achromobacter xylosoxidans A8 | Mobiluncus mulieris ATCC 35243 |
| Achromobacter xylosoxidans AXX-A | Moraxella catarrhalis RH4 |
| Acinetobacter baumannii AB0057 | Mycobacterium tuberculosis CDC1551 |
| Acinetobacter baumannii AB307-0294 | Mycobacterium tuberculosis CPHL A |
| Acinetobacter baumannii AB900 | Mycoplasma fermentans JER |
| Acinetobacter baumannii ACICU | Mycoplasma fermentans M64 |
| Acinetobacter baumannii ATCC 17978 | Mycoplasma hominis |
| Acinetobacter baumannii ATCC 19606 | Mycoplasma pneumoniae M129 |
| Acinetobacter baumannii AYE | Neisseria bacilliformis ATCC BAA-1200 |
| Acinetobacter baumannii SDF | Neisseria elongata subsp glycolytica ATCC 29315 |
| Actinobaculum sp oral taxon 183 str F0552 | Neisseria flavescens NRL30031/H210 |
| Actinomyces cardiffensis F0333 | Neisseria flavescens SK114 |
| Actinomyces georgiae F0490 | Neisseria lactamica 020-06 |
| Actinomyces graevenitzii C83 | Neisseria lactamica ATCC 23970 |
| Actinomyces naeslundii Howell 279 | Neisseria mucosa ATCC 25996 |
| Actinomyces naeslundii MG1 | Neisseria mucosa C102 |
| Actinomyces odontolyticus ATCC 17982 | Neisseria polysaccharea ATCC 43768 |
| Actinomyces odontolyticus F0309 | Neisseria polysaccharea NS342 |
| Actinomyces oris K20 | Neisseria sicca ATCC 29256 |
| Actinomyces sp oral taxon 170 str F0386 | Neisseria sicca DS1 |
| Actinomyces sp oral taxon 171 str F0337 | Neisseria sp oral taxon 014 str F0314 |
| Actinomyces sp oral taxon 172 str F0311 | Neisseria sp oral taxon 020 str F0370 |
| Actinomyces sp oral taxon 175 str F0384 | Neisseria subflava NJ9703 |
| Actinomyces sp oral taxon 178 str F0338 | Neisseria weaveri ATCC 51223 |
| Actinomyces sp oral taxon 180 str F0310 | Ochrobactrum anthropi ATCC 49188 |
| Actinomyces sp oral taxon 181 str F0379 | Olsenella sp oral taxon 809 str F0356 |
| Actinomyces sp oral taxon 448 str F0400 | Olsenella uli DSM 7084 |
| Actinomyces sp oral taxon 848 str F0332 | Oribacterium sinus F0268 |
| Actinomyces sp oral taxon 849 str F0330 | Oribacterium sp oral taxon 078 str F0262 |
| Actinomyces viscosus C505 | Oribacterium sp oral taxon 108 str F0425 |
| Afipia broomeae ATCC 49717 | Paenibacillus sp oral taxon 786 str D14 |
| Afipia sp 1NLS2 | Parascardovia denticolens DSM 10105 |
| Aggregatibacter actinomycetemcomitans ANH9381 | Parascardovia denticolens F0305 |
| Aggregatibacter actinomycetemcomitans D11S-1 | Parvimonas micra ATCC 33270 |
| Aggregatibacter actinomycetemcomitans D17P-2 | Parvimonas sp oral taxon 110 str F0139 |
| Aggregatibacter actinomycetemcomitans D17P-3 | Parvimonas sp oral taxon 393 str F0440 |
| Aggregatibacter actinomycetemcomitans D7S-1 | Peptoniphilus indolicus ATCC 29427 |
| Aggregatibacter actinomycetemcomitans RhAA1 | Peptoniphilus lacrimalis 315-B |
| Aggregatibacter actinomycetemcomitans serotype a str H5P1 | Peptoniphilus sp oral taxon 375 str F0436 |
| Aggregatibacter actinomycetemcomitans serotype b str I23C | Peptoniphilus sp oral taxon 386 str F0131 |
| Aggregatibacter actinomycetemcomitans serotype b str SCC1398 | Peptoniphilus sp oral taxon 836 str F0141 |
| Aggregatibacter actinomycetemcomitans serotype c str SCC2302 | Peptostreptococcus anaerobius 653-L |
| Aggregatibacter actinomycetemcomitans serotype d str I63B | Peptostreptococcus stomatis DSM 17678 |
| Aggregatibacter actinomycetemcomitans serotype e str SC1083 | Porphyromonas asaccharolytica DSM 20707 |
| Aggregatibacter actinomycetemcomitans serotype e str SCC393 | Porphyromonas asaccharolytica PR426713P-I |
| Aggregatibacter actinomycetemcomitans serotype f str D18P1 | Porphyromonas catoniae F0037 |
| Aggregatibacter aphrophilus NJ8700 | Porphyromonas endodontalis ATCC 35406 |
| Aggregatibacter segnis ATCC 33393 | Porphyromonas gingivalis ATCC 33277 |
| Aggregatibacter sp oral taxon 458 str W10330 | Porphyromonas gingivalis TDC60 |
| Alloscardovia omnicolens F0580 | Porphyromonas gingivalis W50 |
| Anaerococcus lactolyticus ATCC 51172 | Porphyromonas gingivalis W83 |
| Anaerococcus prevotii ACS-065-V-Col13 | Porphyromonas sp oral taxon 278 str W7784 |
| Anaerococcus prevotii DSM 20548 | Porphyromonas sp oral taxon 279 str F0450 |
| Anaerococcus tetradius ATCC 35098 | Prevotella bivia JCVIHMP010 |
| Anaeroglobus geminatus F0357 | Prevotella buccae ATCC 33574 |
| Arcanobacterium haemolyticum DSM 20595 | Prevotella buccae D17 |
| Atopobium minutum 10063974 | Prevotella buccalis ATCC 35310 |
| Atopobium parvulum DSM 20469 | Prevotella dentalis DSM 3688 |
| Atopobium rimae ATCC 49626 | Prevotella denticola CRIS 18C-A |
| Atopobium sp oral taxon 199 str F0494 | Prevotella denticola F0289 |
| Atopobium sp oral taxon 810 str F0209 | Prevotella histicola F0411 |
| Bacillus clausii KSM-K16 | Prevotella intermedia 17 |
| Bacillus subtilis subsp spizizenii str W23 | Prevotella loescheii DSM 19665 |
| Bacillus subtilis subsp subtilis str NCIB 3610 | Prevotella maculosa OT 289 |
| Bacillus subtilis subsp subtilis str SMY | Prevotella marshii DSM 16973 |
| Bacteroidetes oral taxon 272 str F0290 | Prevotella melaninogenica ATCC 25845 |
| Bacteroidetes oral taxon 274 str F0058 | Prevotella melaninogenica D18 |
| Bifidobacterium animalis subsp lactis AD011 | Prevotella micans F0438 |
| Bifidobacterium breve DPC 6330 | Prevotella multiformis DSM 16608 |
| Bifidobacterium breve UCC2003 | Prevotella multisaccharivorax DSM 17128 |
| Bifidobacterium dentium ATCC 27678 | Prevotella nigrescens ATCC 33563 |
| Bifidobacterium dentium ATCC 27679 | Prevotella oralis ATCC 33269 |
| Bifidobacterium dentium Bd1 | Prevotella oris C735 |
| Bifidobacterium dentium JCVIHMP022 | Prevotella oris F0302 |
| Bifidobacterium longum subsp infantis ATCC 15697 | Prevotella oulorum F0390 |
| Bifidobacterium longum subsp longum BBMN68 | Prevotella pallens ATCC 700821 |
| Brevundimonas diminuta ATCC 11568 | Prevotella saccharolytica F0055 |
| Bulleidia extructa W1219 | Prevotella salivae DSM 15606 |
| Burkholderia cepacia GG4 | Prevotella sp oral taxon 299 str F0039 |
| Campylobacter concisus 13826 | Prevotella sp oral taxon 302 str F0323 |
| Campylobacter curvus 52592 | Prevotella sp oral taxon 306 str F0472 |
| Campylobacter gracilis RM3268 | Prevotella sp oral taxon 317 str F0108 |
| Campylobacter rectus RM3267 | Prevotella sp oral taxon 472 str F0295 |
| Campylobacter showae RM3277 | Prevotella sp oral taxon 473 str F0040 |
| candidate division SR1 bacterium MGEHA | Prevotella tannerae ATCC 51259 |
| candidate division TM7 genomosp GTL1 | Prevotella veroralis F0319 |
| candidate division TM7 single-cell isolate TM7a | Propionibacterium acnes J139 |
| candidate division TM7 single-cell isolate TM7b | Propionibacterium acnes KPA171202 |
| candidate division TM7 single-cell isolate TM7c | Propionibacterium acnes SK137 |
| Capnocytophaga gingivalis ATCC 33624 | Propionibacterium avidum ATCC 25577 |
| Capnocytophaga granulosa ATCC 51502 | Propionibacterium propionicum F0230a |
| Capnocytophaga ochracea DSM 7271 | Propionibacterium sp oral taxon 192 str F0372 |
| Capnocytophaga ochracea F0287 | Proteus mirabilis ATCC 29906 |
| Capnocytophaga sp oral taxon 324 str F0483 | Proteus mirabilis HI4320 |
| Capnocytophaga sp oral taxon 326 str F0382 | Pseudomonas aeruginosa LESB58 |
| Capnocytophaga sp oral taxon 329 str F0087 | Pseudomonas aeruginosa PA7 |
| Capnocytophaga sp oral taxon 332 str F0381 | Pseudomonas aeruginosa UCBPP-PA14 |
| Capnocytophaga sp oral taxon 335 str F0486 | Pseudomonas fluorescens Pf-5 |
| Capnocytophaga sp oral taxon 336 str F0502 | Pseudomonas fluorescens Pf0-1 |
| Capnocytophaga sp oral taxon 338 str F0234 | Pseudomonas fluorescens SBW25 |
| Capnocytophaga sp oral taxon 380 str F0488 | Pseudomonas pseudoalcaligenes KF707 |
| Capnocytophaga sp oral taxon 412 str F0487 | Pseudomonas stutzeri A1501 |
| Capnocytophaga sp oral taxon 863 str F0517 | Pseudomonas stutzeri ATCC 17588 = LMG 11199 |
| Capnocytophaga sputigena ATCC 33612 | Pseudoramibacter alactolyticus ATCC 23263 |
| Cardiobacterium hominis ATCC 15826 | Pyramidobacter piscolens W5455 |
| Cardiobacterium valvarum F0432 | Ralstonia pickettii 12D |
| Catonella morbi ATCC 51271 | Rhodobacter capsulatus SB1003 |
| Centipeda periodontii DSM 2778 | Rothia aeria F0474 |
| Chlamydophila pneumoniae AR39 | Rothia dentocariosa ATCC 17931 |
| Chlamydophila pneumoniae CWL029 | Rothia dentocariosa M567 |
| Chlamydophila pneumoniae J138 | Rothia mucilaginosa ATCC 25296 |
| Chlamydophila pneumoniae TW-183 | Rothia mucilaginosa DY-18 |
| Comamonas testosteroni ATCC 11996 | Rothia sp oral taxon 188 str F0184 |
| Comamonas testosteroni NBRC 100989 | Sanguibacter keddieii DSM 10542 |
| Corynebacterium matruchotii ATCC 14266 | Scardovia inopinata F0304 |
| Corynebacterium matruchotii ATCC 33806 | Scardovia wiggsiae F0424 |
| Corynebacterium urealyticum DSM 7109 | Selenomonas artemidis F0399 |
| Cronobacter sakazakii ATCC BAA-894 | Selenomonas flueggei ATCC 43531 |
| Cronobacter sakazakii E899 | Selenomonas infelix ATCC 43532 |
| Cryptobacterium curtum DSM 15641 | Selenomonas noxia ATCC 43541 |
| Delftia acidovorans SPH-1 | Selenomonas noxia F0398 |
| Desulfobulbus sp oral taxon 041 str Dsb1-5 | Selenomonas sp oral taxon 137 str F0430 |
| Desulfobulbus sp oral taxon 041 str Dsb2 | Selenomonas sp oral taxon 138 str F0429 |
| Desulfobulbus sp oral taxon 041 str Dsb4 | Selenomonas sp oral taxon 149 str 67H29BP |
| Dialister invisus DSM 15470 | Selenomonas sp oral taxon 892 str F0426 |
| Dialister micraerophilus DSM 19965 | Selenomonas sputigena ATCC 35185 |
| Dialister microaerophilus UPII 345-E | Shuttleworthia satelles DSM 14600 |
| Dolosigranulum pigrum ATCC 51524 | Simonsiella muelleri ATCC 29453 |
| Eggerthella lenta DSM 2243 | Slackia exigua ATCC 700122 |
| Eikenella corrodens ATCC 23834 | Solobacterium moorei F0204 |
| Enterobacter cancerogenus ATCC 35316 | Staphylococcus aureus A5937 |
| Enterobacter hormaechei ATCC 49162 | Staphylococcus aureus subsp aureus ATCC 51811 |
| Enterococcus casseliflavus EC10 | Staphylococcus aureus subsp aureus ATCC BAA-39 |
| Enterococcus casseliflavus EC20 | Staphylococcus caprae C87 |
| Enterococcus casseliflavus EC30 | Staphylococcus epidermidis ATCC 12228 |
| Enterococcus durans ATCC 6056 | Staphylococcus epidermidis BCM-HMP0060 |
| Enterococcus faecalis ARO1 DG | Staphylococcus warneri L37603 |
| Enterococcus faecalis ATCC 29200 | Stenotrophomonas maltophilia K279a |
| Enterococcus faecalis ATCC 4200 | Stenotrophomonas maltophilia R551-3 |
| Enterococcus italicus DSM 15952 | Stenotrophomonas sp SKA14 |
| Enterococcus saccharolyticus 30 1 | Streptococcus agalactiae 2603VR |
| Eubacterium infirmum F0142 | Streptococcus agalactiae 515 |
| Eubacterium limosum KIST612 | Streptococcus agalactiae A909 |
| Eubacterium nodatum ATCC 33099 | Streptococcus agalactiae CJB111 |
| Eubacterium saburreum DSM 3986 | Streptococcus agalactiae COH1 |
| Eubacterium saphenum ATCC 49989 | Streptococcus agalactiae H36B |
| Eubacterium yurii subsp margaretiae ATCC 43715 | Streptococcus agalactiae NEM316 |
| Filifactor alocis ATCC 35896 | Streptococcus anginosus 1 2 62CV |
| Finegoldia magna ATCC 29328 | Streptococcus anginosus F0211 |
| Finegoldia magna ATCC 53516 | Streptococcus australis ATCC 700641 |
| Finegoldia magna BVS033A4 | Streptococcus constellatus subsp constellatus SK53 |
| Fusobacterium gonidiaformans ATCC 25563 | Streptococcus cristatus ATCC 51100 |
| Fusobacterium necrophorum subsp funduliforme ATCC 51357 | Streptococcus downei F0415 |
| Fusobacterium nucleatum subsp fusiforme ATCC 51190 | Streptococcus gordonii str Challis substr CH1 |
| Fusobacterium nucleatum subsp nucleatum ATCC 23726 | Streptococcus infantarius subsp infantarius ATCC BAA-102 |
| Fusobacterium nucleatum subsp nucleatum ATCC 25586 | Streptococcus infantis ATCC 700779 |
| Fusobacterium nucleatum subsp polymorphum ATCC 10953 | Streptococcus infantis SK1302 |
| Fusobacterium nucleatum subsp polymorphum F0401 | Streptococcus intermedius F0395 |
| Fusobacterium nucleatum subsp vincentii ATCC 49256 | Streptococcus intermedius F0413 |
| Fusobacterium periodonticum ATCC 33693 | Streptococcus intermedius JTH08 |
| Fusobacterium periodonticum D10 | Streptococcus intermedius SK54 |
| Fusobacterium sp oral taxon 370 str F0437 | Streptococcus mitis ATCC 6249 |
| Gemella haemolysans ATCC 10379 | Streptococcus mitis B6 |
| Gemella haemolysans M341 | Streptococcus mitis bv 2 str F0392 |
| Gemella moribillum M424 | Streptococcus mitis bv 2 str SK95 |
| Gemella sanguinis M325 | Streptococcus mitis NCTC 12261 |
| Granulicatella adiacens ATCC 49175 | Streptococcus mitis SK1073 |
| Granulicatella elegans ATCC 700633 | Streptococcus mitis SK1080 |
| Haemophilus aegyptius ATCC 11116 | Streptococcus mitis SK321 |
| Haemophilus ducreyi 35000HP | Streptococcus mitis SK564 |
| Haemophilus haemolyticus M19107 | Streptococcus mitis SK569 |
| Haemophilus haemolyticus M21639 | Streptococcus mitis SK575 |
| Haemophilus sp oral taxon 851 str F0397 | Streptococcus mitis SK579 |
| Helicobacter pylori B38 | Streptococcus mitis SK597 |
| Johnsonella ignava ATCC 51276 | Streptococcus mitis SK616 |
| Jonquetella anthropi DSM 22815 | Streptococcus mitis SPAR10 |
| Kingella denitrificans ATCC 33394 | Streptococcus mutans GS-5 |
| Kingella kingae ATCC 23330 | Streptococcus mutans NN2025 |
| Kingella oralis ATCC 51147 | Streptococcus mutans TCI-101 |
| Klebsiella pneumoniae subsp rhinoscleromatis ATCC 13884 | Streptococcus mutans TCI-109 |
| Kytococcus sedentarius DSM 20547 | Streptococcus mutans TCI-11 |
| Lachnospiraceae bacterium oral taxon 082 str F0431 | Streptococcus mutans TCI-110 |
| Lachnospiraceae oral taxon 107 str F0167 | Streptococcus mutans TCI-116 |
| Lactobacillus acidophilus ATCC 4796 | Streptococcus mutans TCI-239 |
| Lactobacillus acidophilus NCFM | Streptococcus mutans TCI-242 |
| Lactobacillus brevis ATCC 367 | Streptococcus mutans TCI-243 |
| Lactobacillus brevis subsp gravesensis ATCC 27305 | Streptococcus mutans UA159 |
| Lactobacillus buchneri ATCC 11577 | Streptococcus oligofermentans AS 13089 |
| Lactobacillus buchneri NRRL B-30929 | Streptococcus oralis ATCC 35037 |
| Lactobacillus casei ATCC 334 | Streptococcus oralis SK10 |
| Lactobacillus casei BL23 | Streptococcus oralis SK100 |
| Lactobacillus casei str Zhang | Streptococcus oralis SK1074 |
| Lactobacillus coleohominis 101-4-CHN | Streptococcus oralis SK255 |
| Lactobacillus crispatus 125-2-CHN | Streptococcus oralis SK304 |
| Lactobacillus crispatus JV-V01 | Streptococcus oralis SK313 |
| Lactobacillus crispatus MV-1A-US | Streptococcus oralis SK610 |
| Lactobacillus crispatus MV-3A-US | Streptococcus oralis Uo5 |
| Lactobacillus fermentum 28-3-CHN | Streptococcus parasanguinis ATCC 15912 |
| Lactobacillus fermentum ATCC 14931 | Streptococcus parasanguinis ATCC 903 |
| Lactobacillus gasseri 202-4 | Streptococcus parasanguinis F0405 |
| Lactobacillus gasseri 224-1 | Streptococcus peroris ATCC 700780 |
| Lactobacillus gasseri ATCC 33323 | Streptococcus pneumoniae 670-6B |
| Lactobacillus gasseri JV-V03 | Streptococcus pneumoniae BS458 |
| Lactobacillus gasseri MV-22 | Streptococcus pneumoniae CCRI 1974M2 |
| Lactobacillus iners AB-1 | Streptococcus pyogenes ATCC 10782 |
| Lactobacillus iners ATCC 55195 | Streptococcus pyogenes M1 GAS |
| Lactobacillus iners DSM 13335 | Streptococcus pyogenes M49 591 |
| Lactobacillus iners SPIN 1401G | Streptococcus salivarius 57I |
| Lactobacillus jensenii 115-3-CHN | Streptococcus salivarius CCHSS3 |
| Lactobacillus jensenii 1153 | Streptococcus salivarius JIM8777 |
| Lactobacillus jensenii JV-V16 | Streptococcus salivarius K12 |
| Lactobacillus jensenii SJ-7A-US | Streptococcus salivarius M18 |
| Lactobacillus johnsonii ATCC 33200 | Streptococcus salivarius PS4 |
| Lactobacillus johnsonii FI9785 | Streptococcus salivarius SK126 |
| Lactobacillus johnsonii NCC 533 | Streptococcus sanguinis ATCC 29667 |
| Lactobacillus kisonensis str F0345 | Streptococcus sanguinis SK36 |
| Lactobacillus oris F0423 | Streptococcus sanguinis VMC66 |
| Lactobacillus oris PB013-T2-3 | Streptococcus sobrinus TCI-107 |
| Lactobacillus paracasei subsp paracasei 8700:2 | Streptococcus sobrinus TCI-118 |
| Lactobacillus paracasei subsp paracasei ATCC 25302 | Streptococcus sobrinus TCI-119 |
| Lactobacillus parafarraginis F0439 | Streptococcus sobrinus TCI-121 |
| Lactobacillus pentosus KCA1 | Streptococcus sobrinus TCI-124 |
| Lactobacillus plantarum JDM1 | Streptococcus sobrinus TCI-157 |
| Lactobacillus plantarum subsp plantarum ATCC 14917 | Streptococcus sobrinus TCI-336 |
| Lactobacillus reuteri 100-23 | Streptococcus sobrinus TCI-345 |
| Lactobacillus reuteri DSM 20016 | Streptococcus sobrinus TCI-349 |
| Lactobacillus reuteri JCM 1112 | Streptococcus sobrinus TCI-355 |
| Lactobacillus reuteri MM2-3 | Streptococcus sobrinus TCI-363 |
| Lactobacillus rhamnosus GG | Streptococcus sobrinus TCI-373 |
| Lactobacillus rhamnosus HN001 | Streptococcus sobrinus TCI-384 |
| Lactobacillus rhamnosus Lc 705 | Streptococcus sobrinus TCI-53 |
| Lactobacillus rhamnosus LMS2-1 | Streptococcus sobrinus TCI-79 |
| Lactobacillus salivarius ACS-116-V-Col5a | Streptococcus sobrinus TCI-9 |
| Lactobacillus salivarius ATCC 11741 | Streptococcus sp oral taxon 056 str F0418 |
| Lactobacillus salivarius CECT 5713 | Streptococcus sp oral taxon 058 str F0407 |
| Lactobacillus salivarius GJ-24 | Streptococcus sp oral taxon 071 str 73H25AP |
| Lactobacillus salivarius NIAS840 | Streptococcus vestibularis ATCC 49124 |
| Lactobacillus salivarius UCC118 | Streptococcus vestibularis F0396 |
| Lactococcus lactis subsp cremoris MG1363 | Synergistetes bacterium SGP1 |
| Lactococcus lactis subsp cremoris SK11 | Tannerella forsythia ATCC 43037 |
| Lactococcus lactis subsp lactis Il1403 | Tannerella sp oral taxon BU063 isolate Cell 1 3 |
| Lactococcus lactis subsp lactis KF147 | Tannerella sp oral taxon BU063 isolate Cell 2 |
| Lautropia mirabilis ATCC 51599 | Tannerella sp oral taxon BU063 isolate Cell 5 |
| Leptotrichia buccalis DSM 1135 | Tannerella sp oral taxon BU063 isolate Cell 6 7 9 |
| Leptotrichia goodfellowii F0264 | Treponema denticola ATCC 35405 |
| Leptotrichia hofstadii F0254 | Treponema denticola F0402 |
| Leptotrichia shahii DSM 19757 | Treponema lecithinolyticum ATCC 700332 |
| Leptotrichia sp oral taxon 215 str W9775 | Treponema maltophilum ATCC 51939 |
| Leptotrichia sp oral taxon 225 str F0581 | Treponema socranskii subsp paredis ATCC 35535 |
| Leptotrichia sp oral taxon 879 str F0557 | Treponema socranskii subsp socranskii VPI DR56BR1116 |
| Leptotrichia wadei DSM 19758 | Treponema vincentii ATCC 35580 |
| Lysinibacillus fusiformis ZC1 | Turicella otitidis ATCC 51513 |
| Megasphaera micronuciformis F0359 | Variovorax paradoxus EPS |
| Mesorhizobium loti MAFF303099 | Variovorax paradoxus S110 |
| Methanobrevibacter smithii ATCC 35061 | Veillonella atypica ACS-049-V-Sch6 |
| Methanobrevibacter smithii DSM 2374 | Veillonella atypica ACS-134-V-Col7a |
| Microbacterium sp oral taxon 186 str F0373 | Veillonella dispar ATCC 17748 |
| Mitsuokella multacida DSM 20544 | Veillonella parvula ACS-068-V-Sch12 |
| Mitsuokella sp oral taxon 131 str W9106 | Veillonella parvula ATCC 17745 |
| Mobiluncus mulieris 28-1 | Veillonella parvula DSM 2008 |
|  | Veillonella sp oral taxon 158 str F0412 |
|  | Veillonella sp oral taxon 780 str F0422 |
