# Supplementary material for: Functional signatures of oral dysbiosis during periodontitis progression revealed by microbial metatranscriptome analysis
Source: Genome Med. 2015 Apr 27;7(1):27. doi: 10.1186/s13073-015-0153-3 (PMC4410737; doi:10.1186/s13073-015-0153-3)
Supplement: Additional file 2: Figure S1. — Statistical differences in metagenome composition. Figure S2. Statistical differences in normalized metatranscriptome composition comparing non-progressing sites baselines to healthy sites of healthy patients. Figure S3. Statistical differences in normalized metatranscriptome composition comparing progressing sites baselines to healthy sites of healthy patients. Figure S4. Venn diagram showing overlapping differentially expressed genes with and without normalization. Figure S5. GO terms associated with changes in gene expression profiles in major periodontal pathogens members of the red complex during periodontal disease progression. Figure S6. GO enrichment analysis comparing healthy sites from healthy individuals and baselines in progressing sites and non-progressing sites. Figure S7. GO terms associated with changes in gene expression profiles in major periodontal pathogens members of the red complex when comparing baselines of progressing and non-progressing sites. Figure S8. GO terms associated with changes in gene expression profiles in members of the orange complex when comparing baselines of progressing and non-progressing sites. Figure S9. GO terms associated with changes in gene expression of putative virulence factors in the oral community during periodontitis progression. Figure S10. GO terms enrichment analysis of virulence factors comparing baselines. Figure S11. GO terms enrichment analysis of virulence factors in the orange complex comparing baselines. Figure S12. Correlation circle plots of sPLS analysis. Figure S13. Percentage of hits corresponding to viral sequences. Figure S14. Statistical differences in viral composition of transcript libraries. [file 13073_2015_153_MOESM2_ESM.pdf]

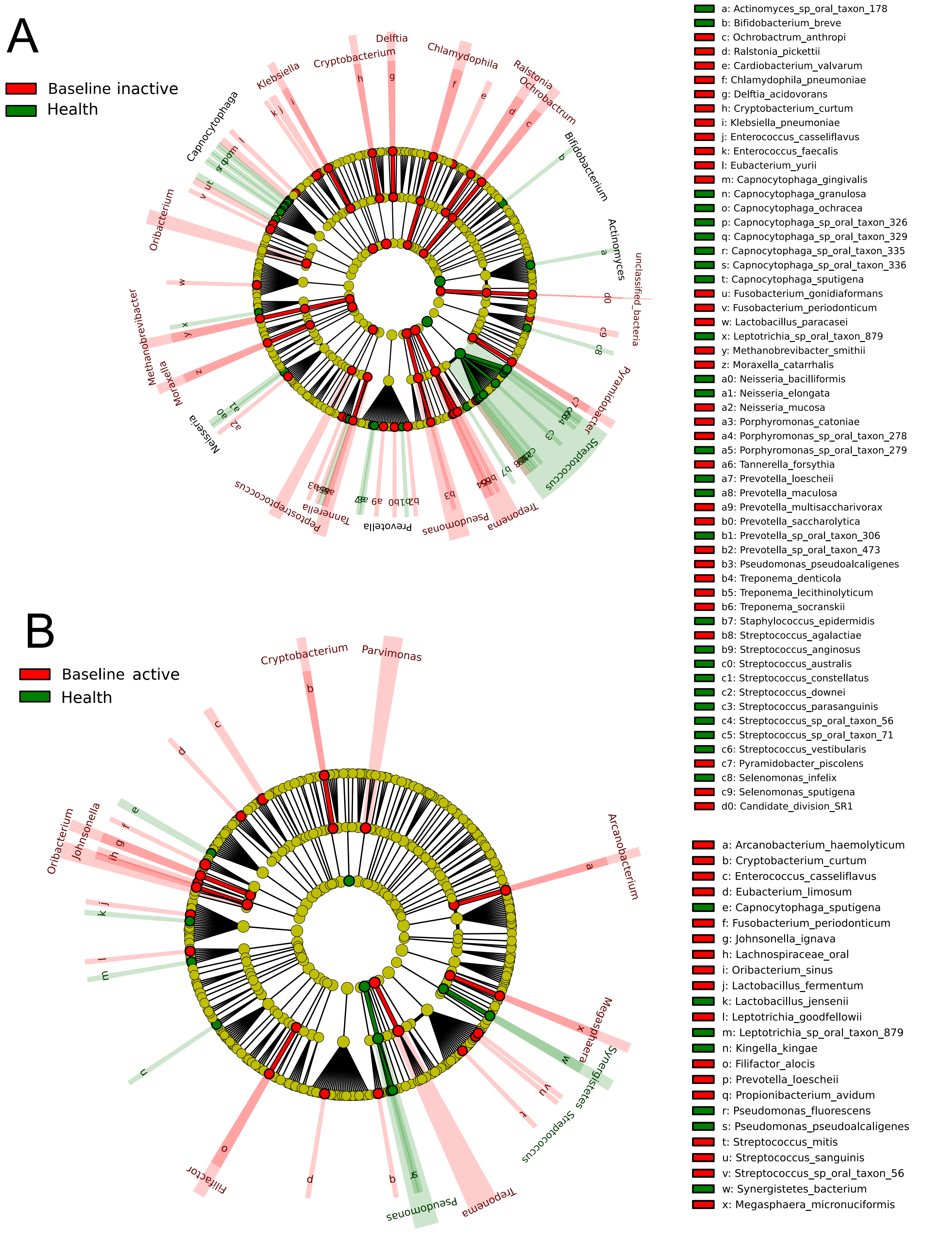

Figure S1. Statistical differences in metagenome composition. Metagenome hit counts were first normalized using GASIC. Normalized counts were then analyzed using LEfSe with default parameters, to identify significant differences at species level between the microbial communities compared.

a) Comparison non-progressing site baselines to healthy sites of healthy patients

b) Comparison progressing site baselines to healthy sites of healthy patients

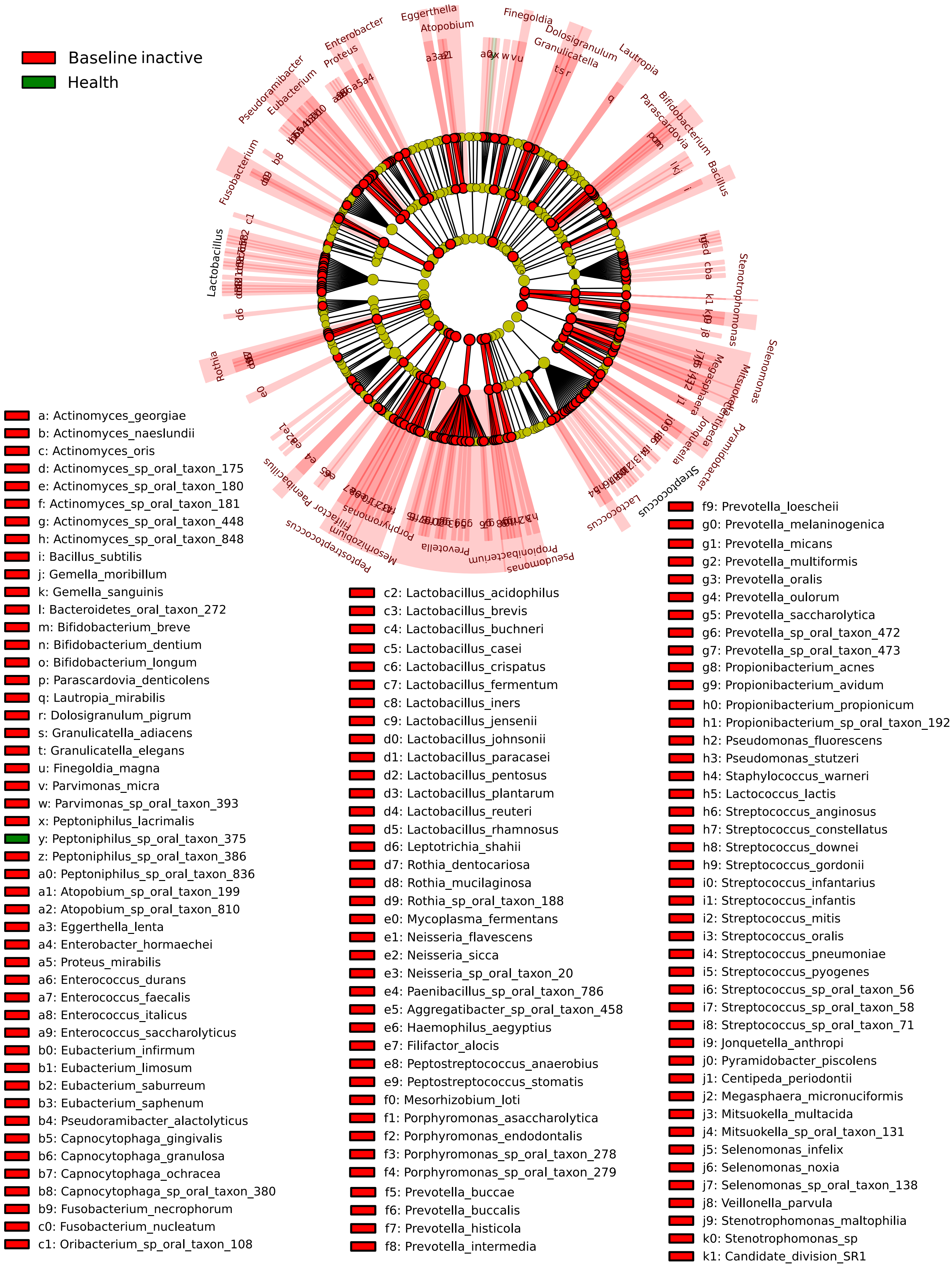

Figure S2. Statistical differences in normalized metatranscriptome composition comparing non-progressing site baselines to healthy sites of healthy patients. Metagenome hit counts were first normalized using GASIC. Metatranscriptome normalized counts were then analyzed using LEfSe with default parameters, to identify significant differences in activity at species level between the microbial communities compared.

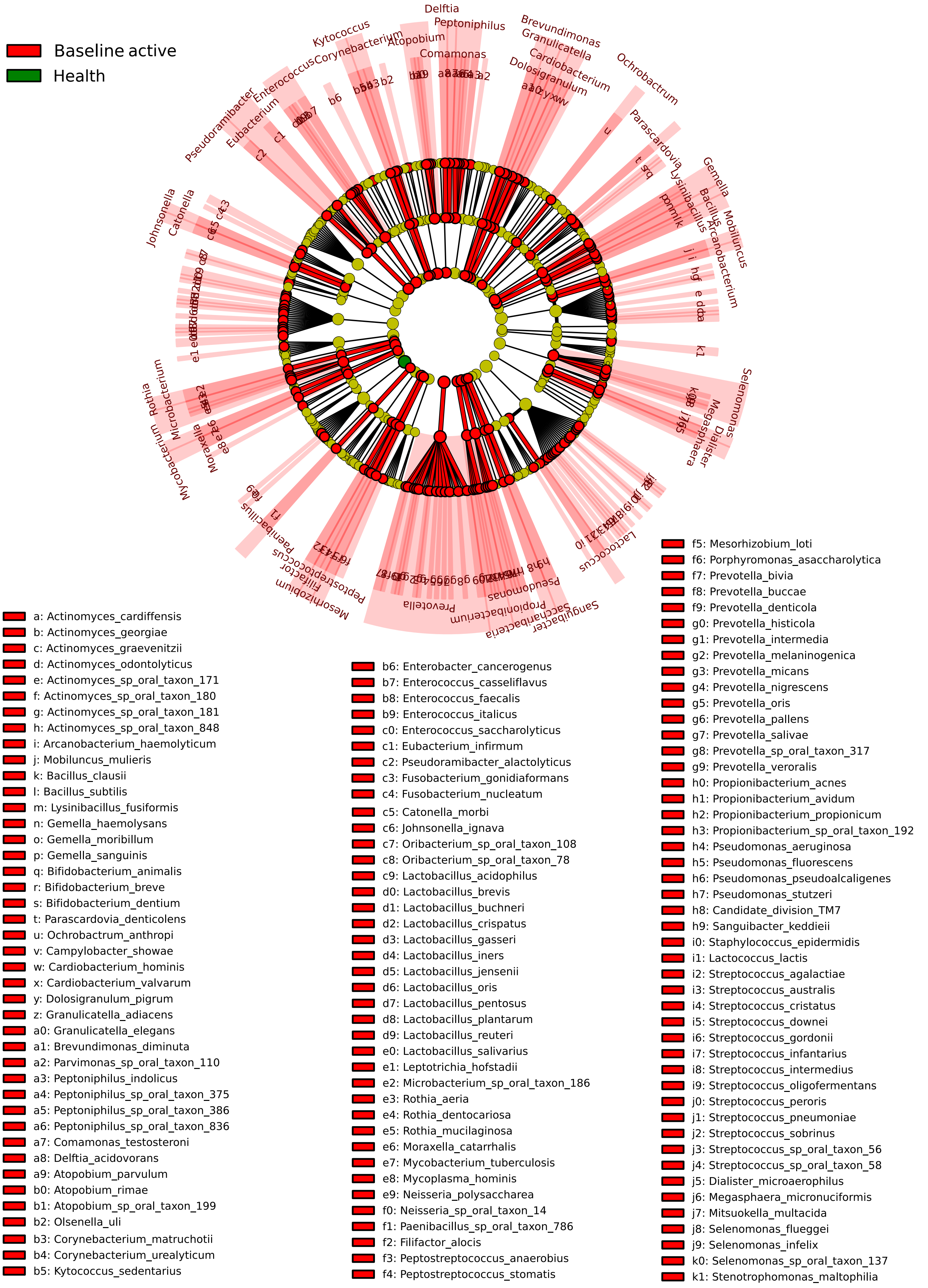

Figure S3. Statistical differences in normalized metatranscriptome composition comparing progressing site baselines to healthy sites of healthy patients. Metagenome hit counts were first normalized using GASIC. Metatranscriptome normalized counts were then analyzed using LEfSe with default parameters, to identify significant differences in activity at species level between the microbial communities compared.

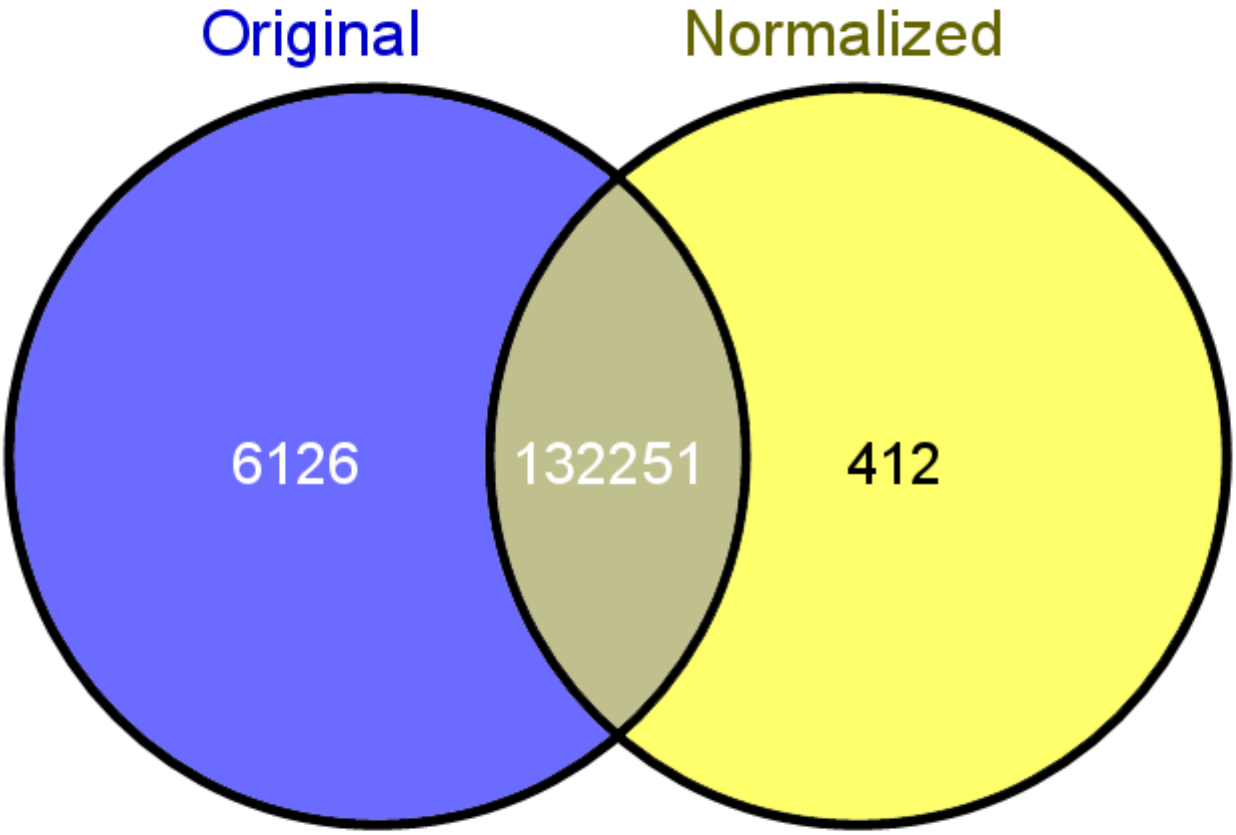

Figure S4. Venn Diagram showing overlaping differentiallly expressed genes comparing baseline and progression with and without normalization. We normalized the expression hit counts against species frequencies estimated using GASIC. Original, differentially expressed genes using raw hits of the metatranscriptome. Venn diagram was obtained using the Venny webpage tool (http://bioinfogp.cnb.csic.es/tools/venny/).

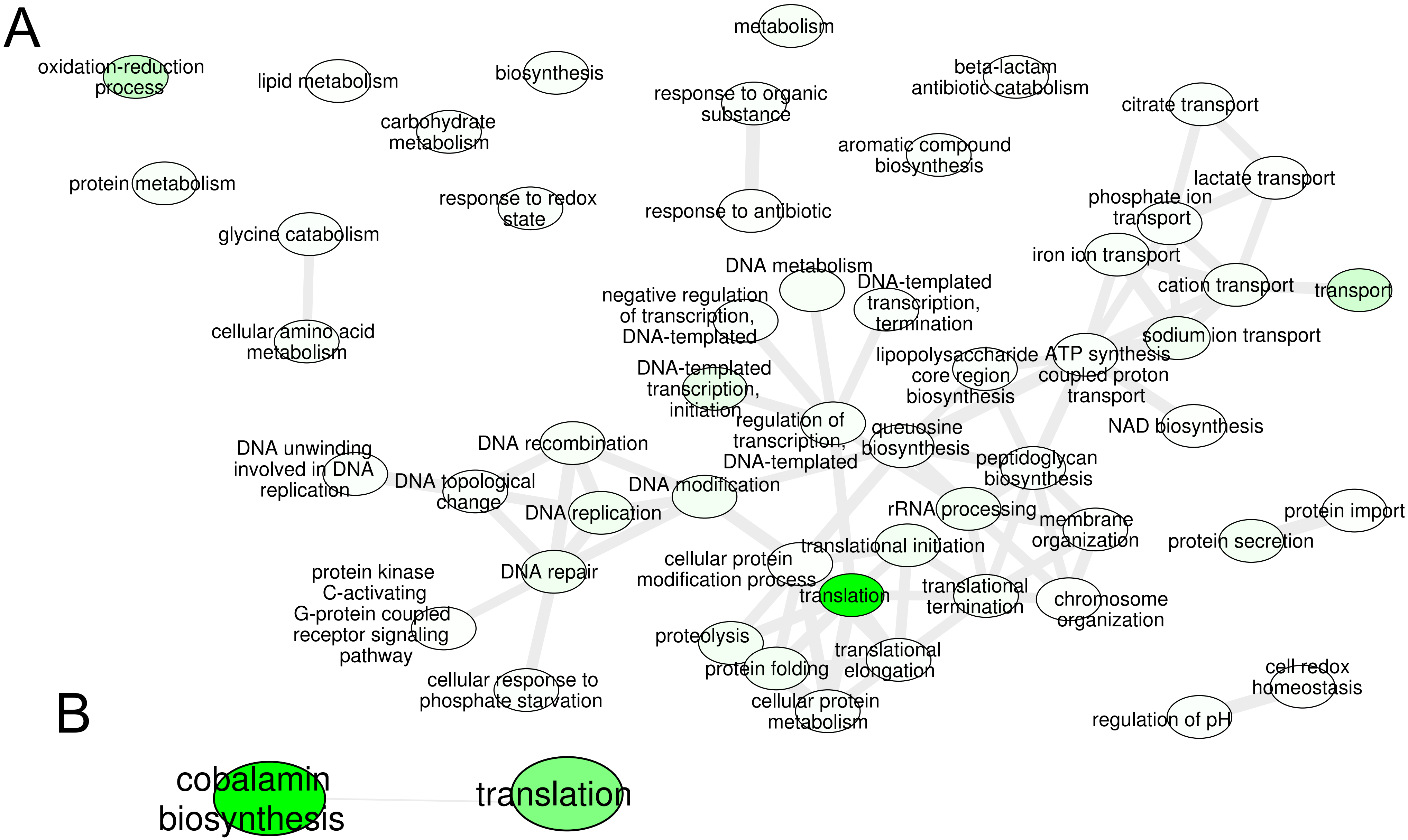

Figure S5. GO terms associated with changes in gene expression profiles in major periodontal pathogens members of the red complex during periodontal disease progression. GO terms were assigned to differentially expressed genes in progression and summaryzed using REVIGO. a) GO terms associated with up-regulated genes in active sites b) GO terms associated with down-regulated genes in active sites.

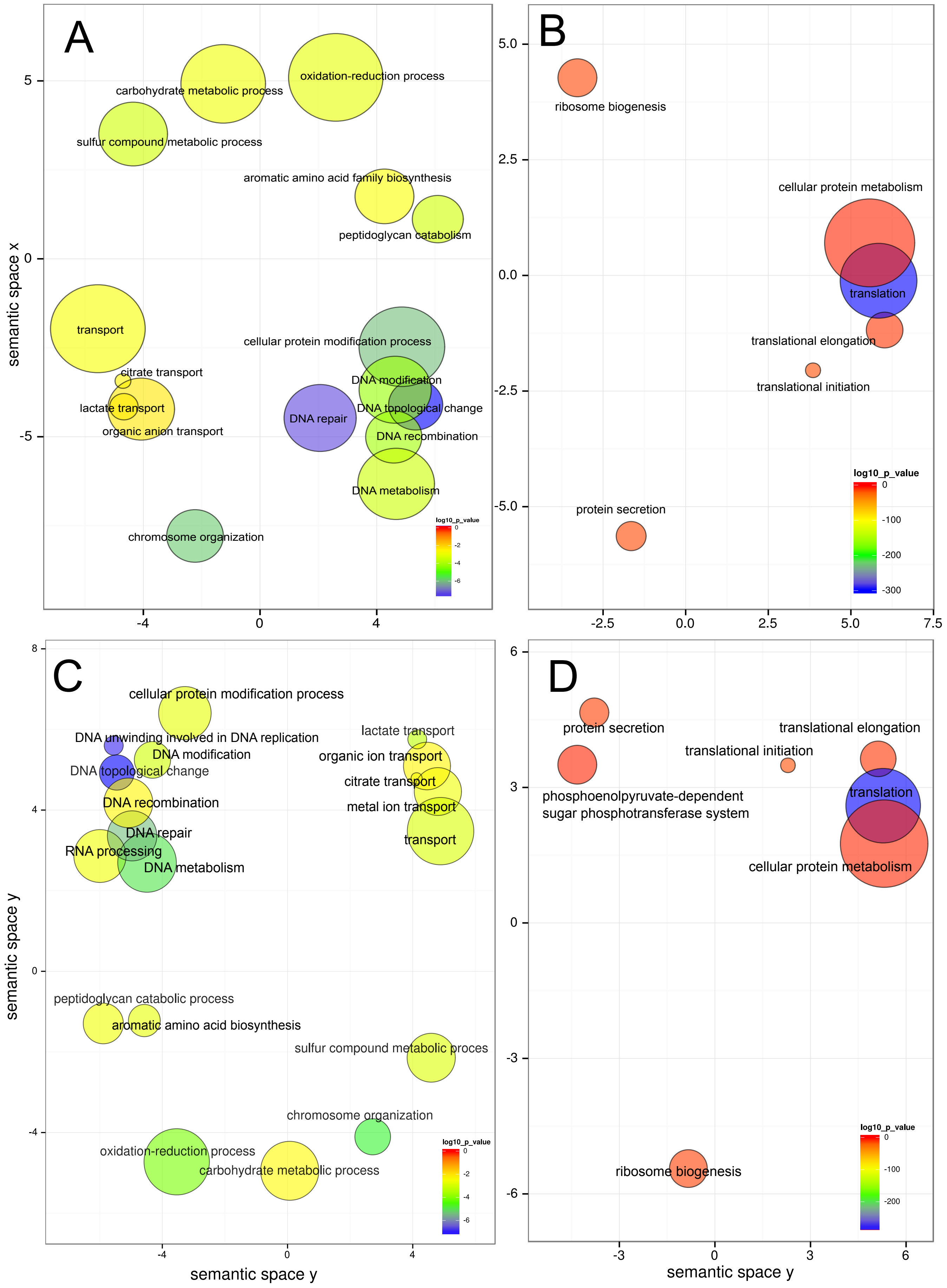

Figure S6. GO enrichment analysis comparing healthy sites from healthy individuals and baselines in progressing and non-progressing sites.. Enriched terms obtained using 'GOseq' were summarized and visualized as a scatter plot using REVIGO. a) Summarized GO terms related to biological processes in inactive baselines.
b) Summarized GO terms related to biological processes in health when compared with inactive baselines.
c) Summarized GO terms related to biological processes in active baselines. d) Summarized GO terms related to biological processes in health when compared with active baselines. Circle size is proportional to the frequency of the GO term, while color indicates the log10 p value (red higher, blue lower). q-value=0.9.

A

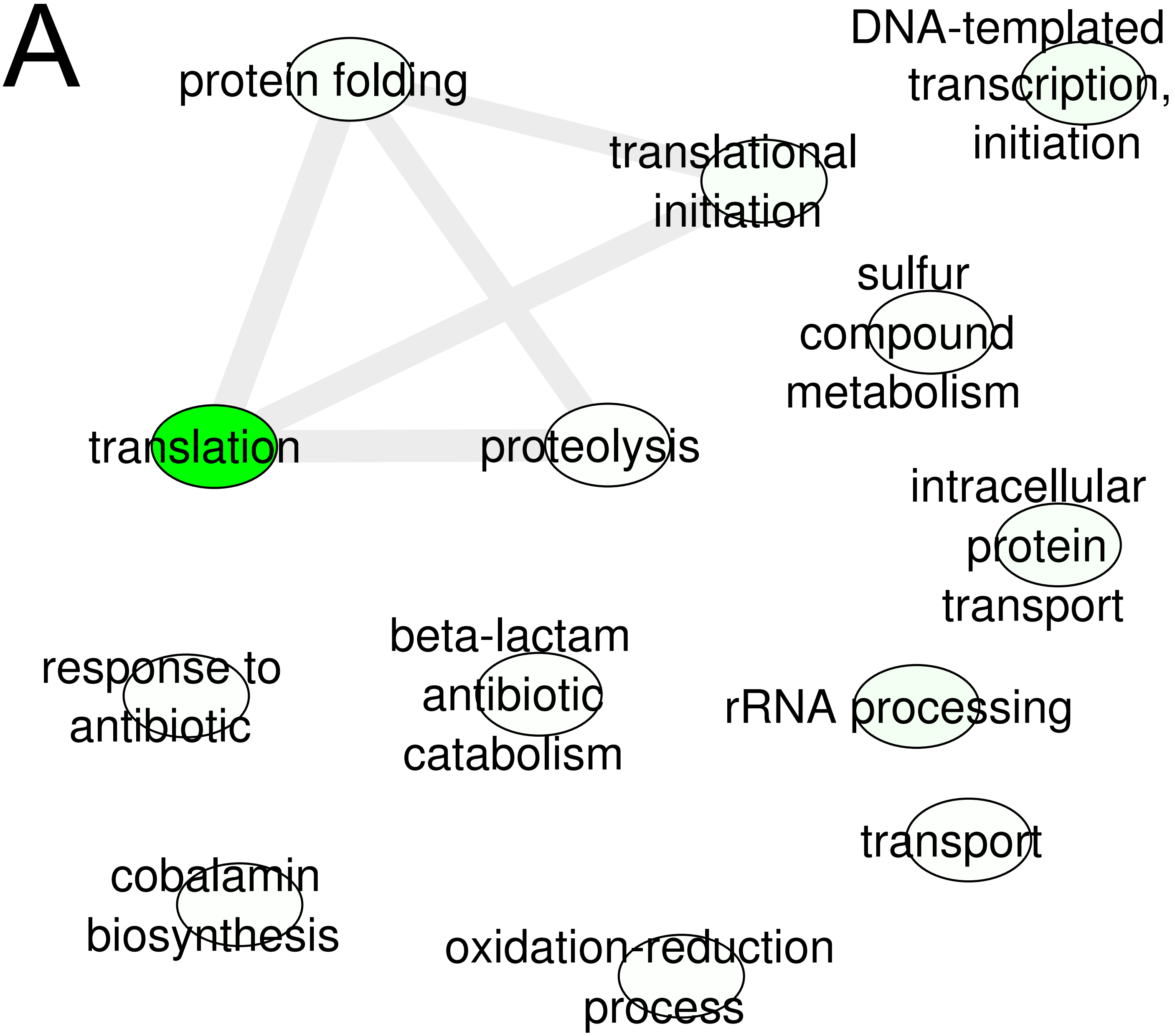

B

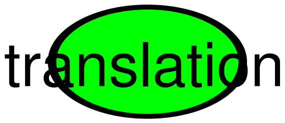

Figure S7. GO terms associated with changes in gene expression profiles in major periodontal pathogens members of the red complex when comparing baselines of active and inactive sites. GO terms were assigned to differentially expressed genes in progression and summaryzed using REVIGO. a) GO terms associated with up-regulated genes in active sites baselines b) GO terms associated with down-regulated genes in active sites baselines.

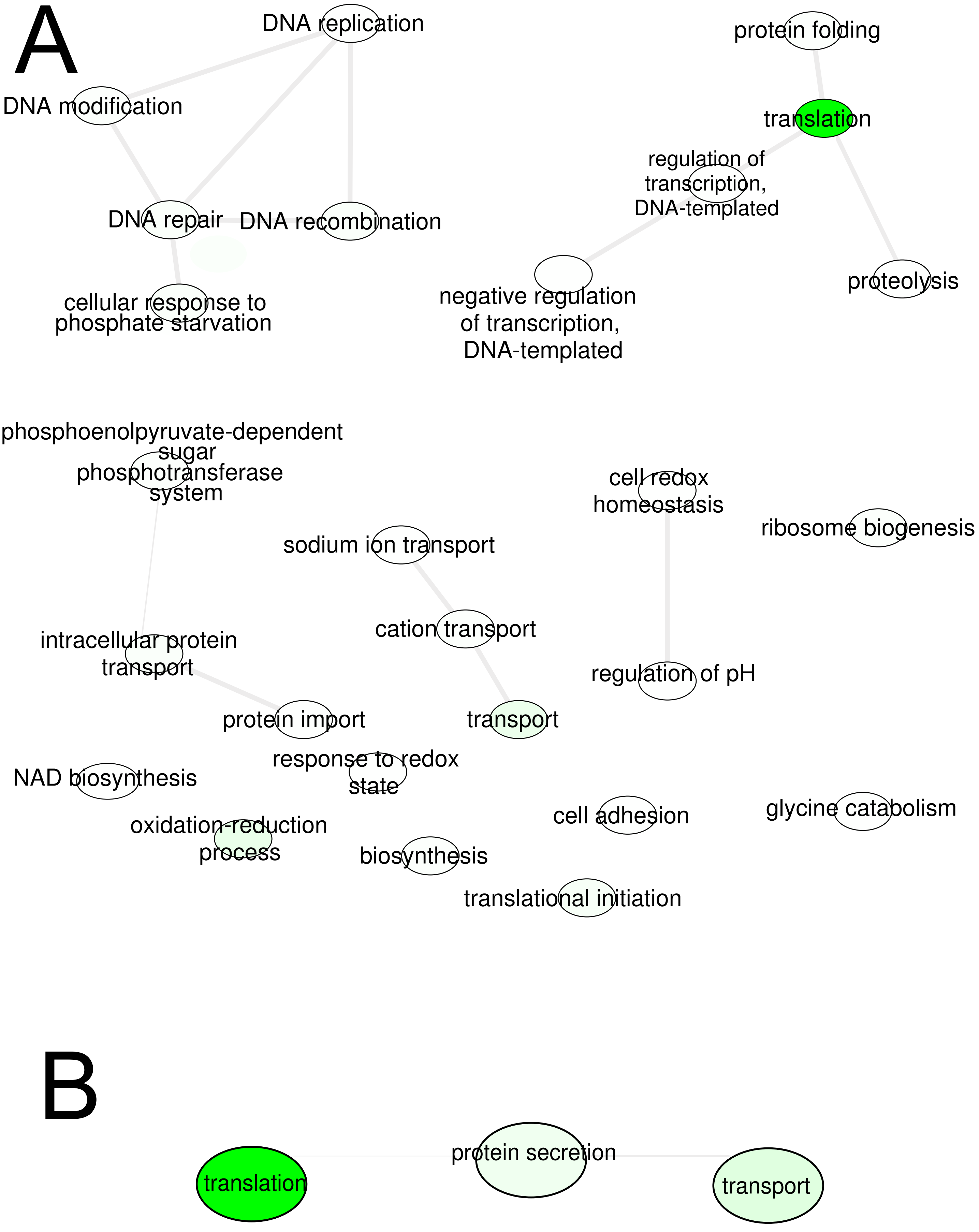

Figure S8. GO terms associated with changes in gene expression profiles in members of the orange complex when comparing baselines of active and inactive sites. GO terms were assigned to differentially expressed genes in progression and summaryzed using REVIGO.
a) GO terms associated with up-regulated genes in active sites baselines b) GO terms associated with down-regulated genes in active sites baselines.

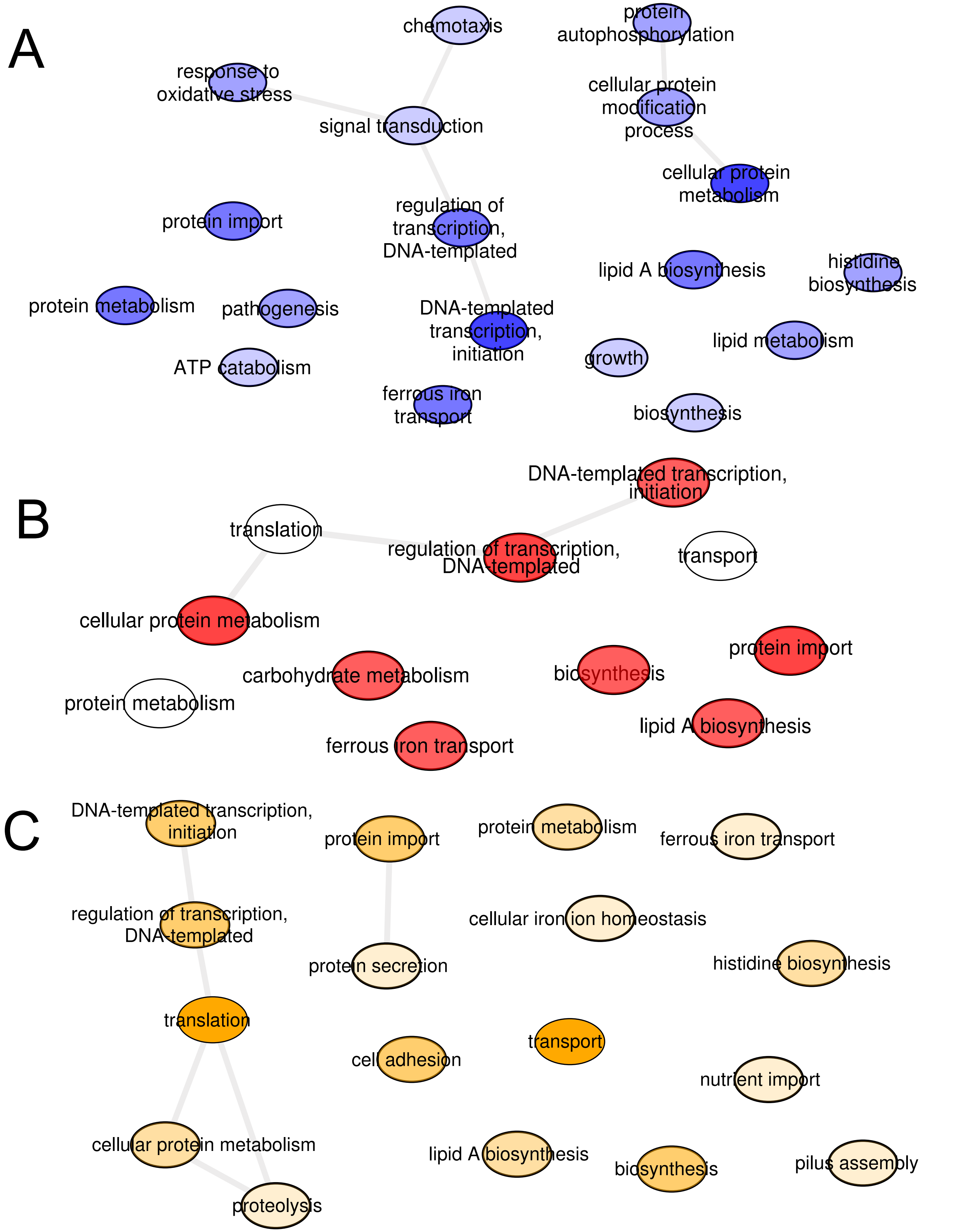

Figure S9. GO terms associated with changes in gene expression of putative virulence factors in the oral community during periodontitis progression. GO terms were assigned to differentially expressed putative virulence factors in progression and summaryzed using REVIGO.
a) GO terms enrichment analysis of virulence factors in the whole community b) GO terms associated with up-regulated virulence factors in the red complex c) GO terms associated with up-regulated virulence factors in the orange complex

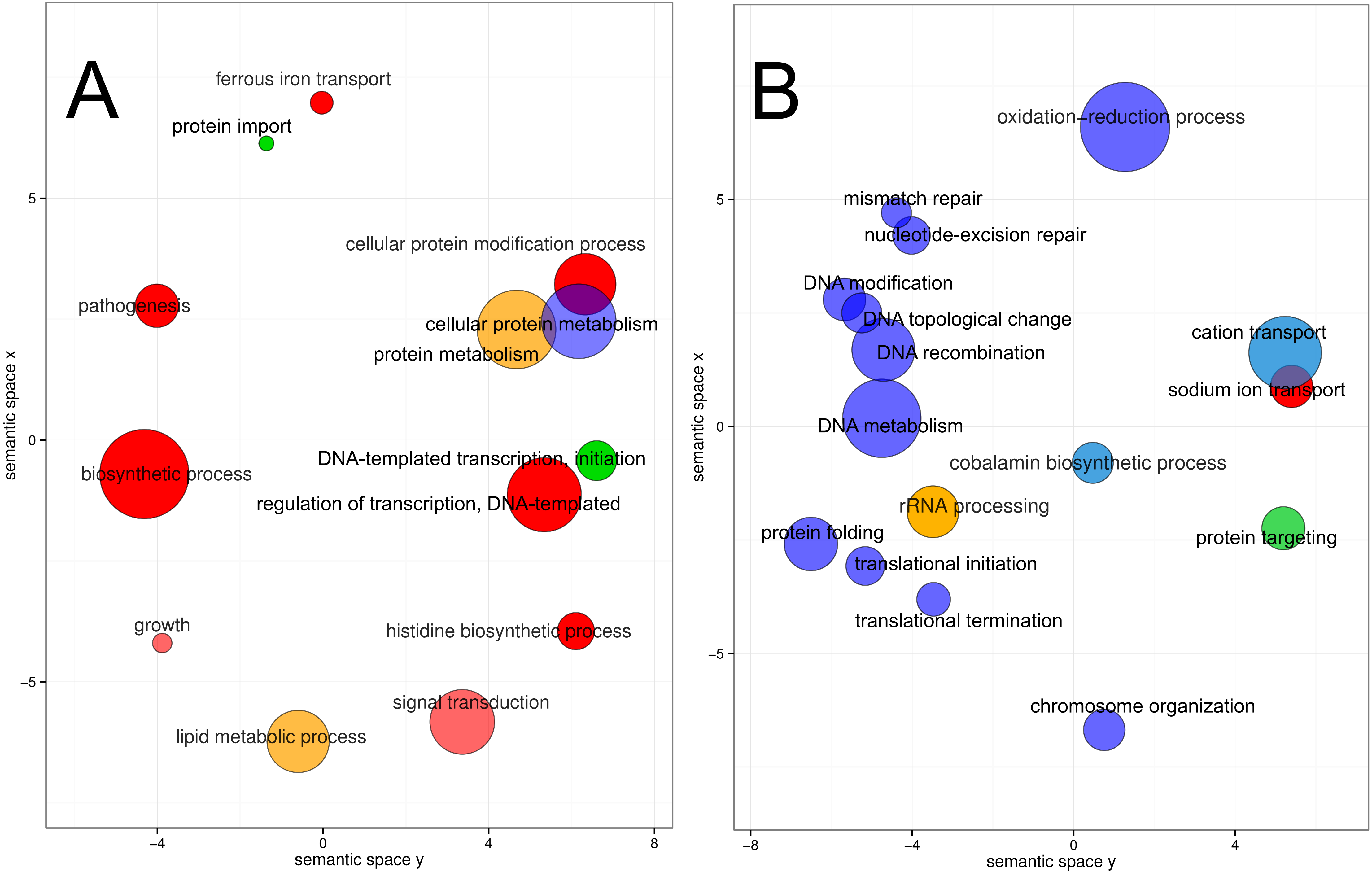

Figure S10. GO terms enrichment analysis of virulence factors comparing baselines. GO terms enrichment was performed using GOseq and summaryzed using REVIGO.

A) GO terms over-represented in progressing sites baselines B) GO terms over-represented in non-progressing sites baselines.

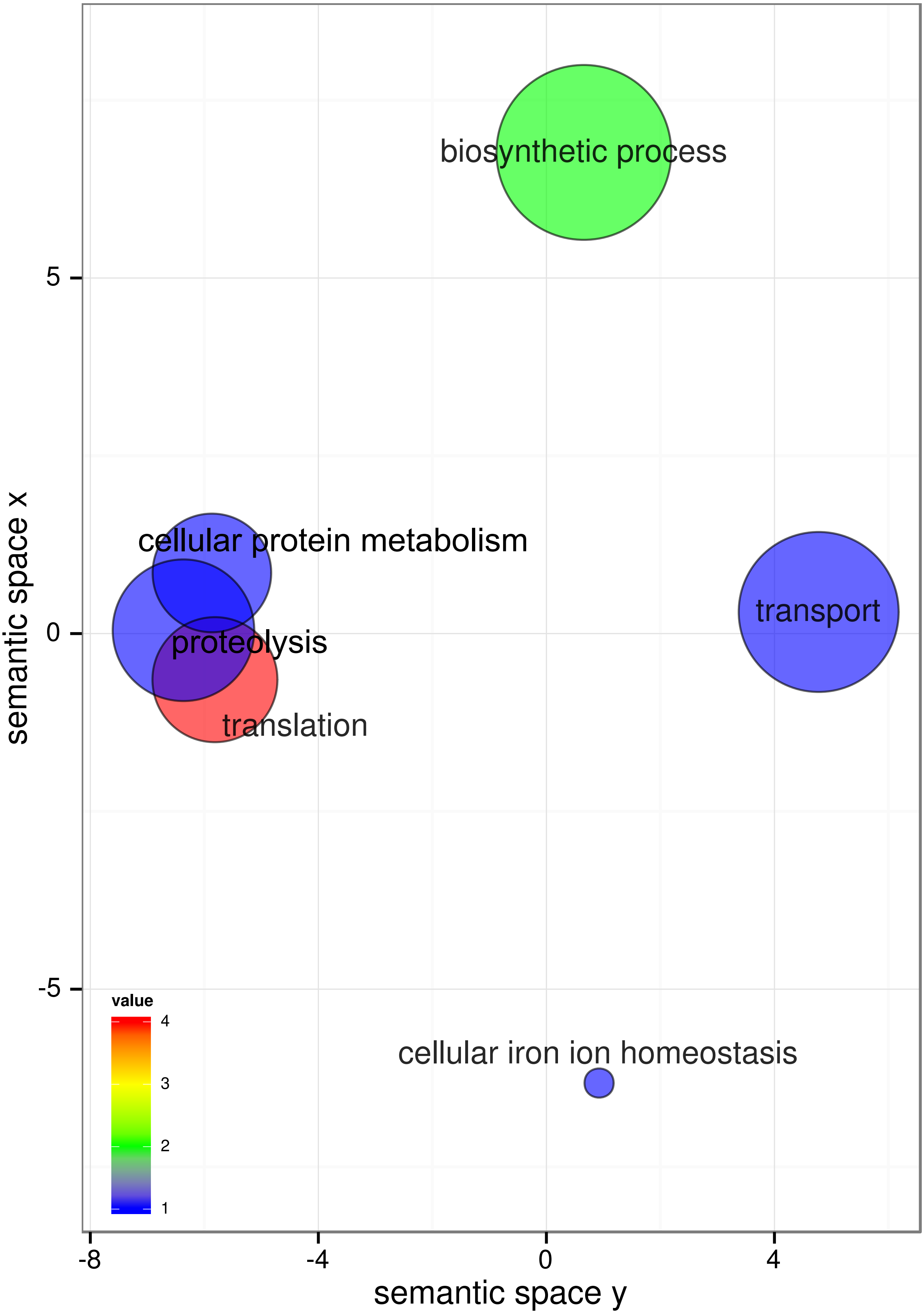

Figure S11. GO terms enrichment analysis of virulence factors in the orange complex comparing baselines. GO terms enrichment was performed using GOseq and summaryzed using REVIGO. GO terms over-represented in baselines of progressing sites.

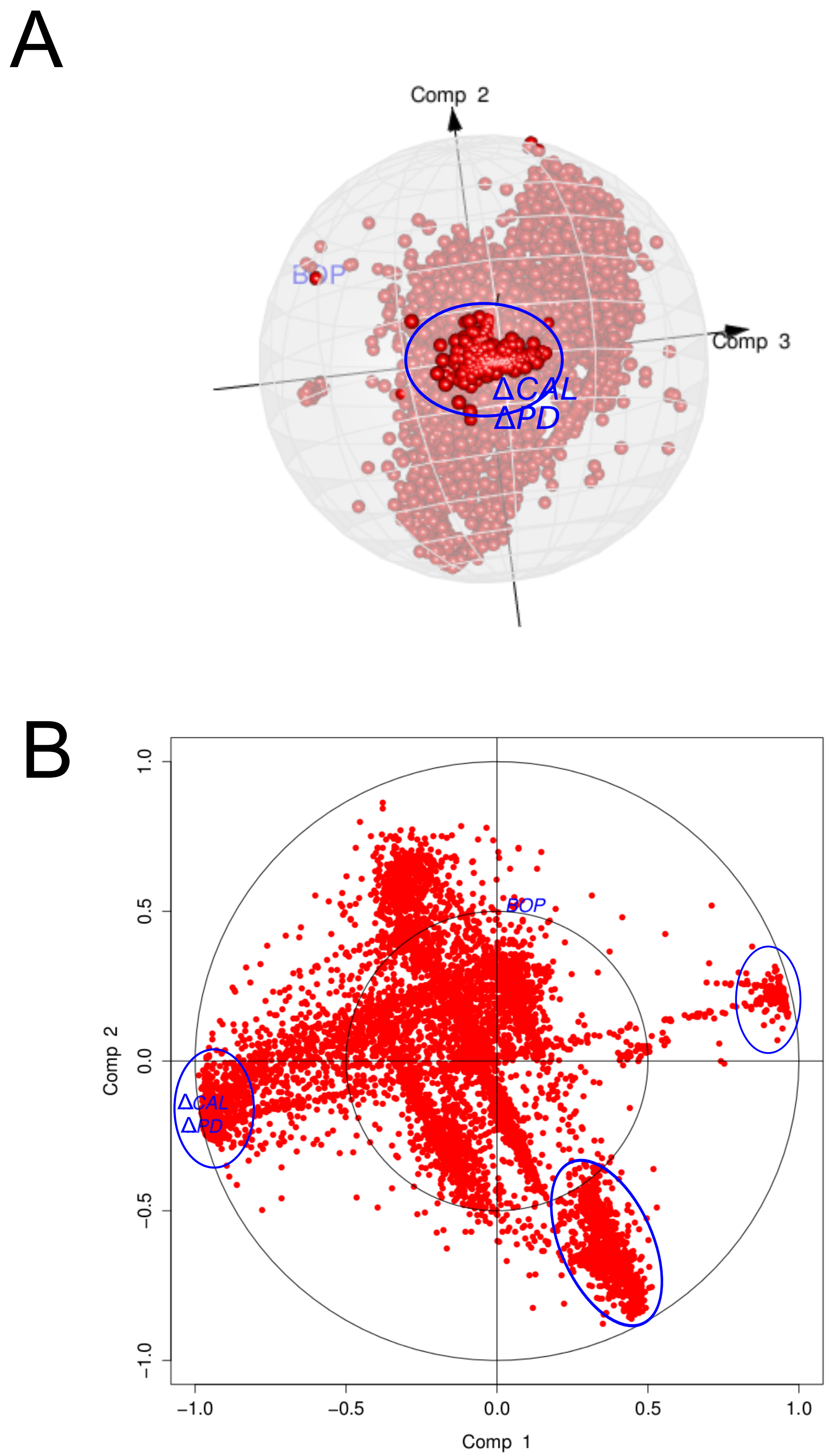

Figure S12. Correlation Circle plots of sPLS analysis. Correlation Circle plots were obtained to assess correlation of the evolution of bleeding on probing (BOP), increase in pocket depth (ΔPD) and increase in clinical attachment level (ΔCAL). A) 3D representation of gene expression associations with evolution of clinical traits (components 1 to 3). B) Gene expression associations with evolution of clinical traits of the 2 first components.

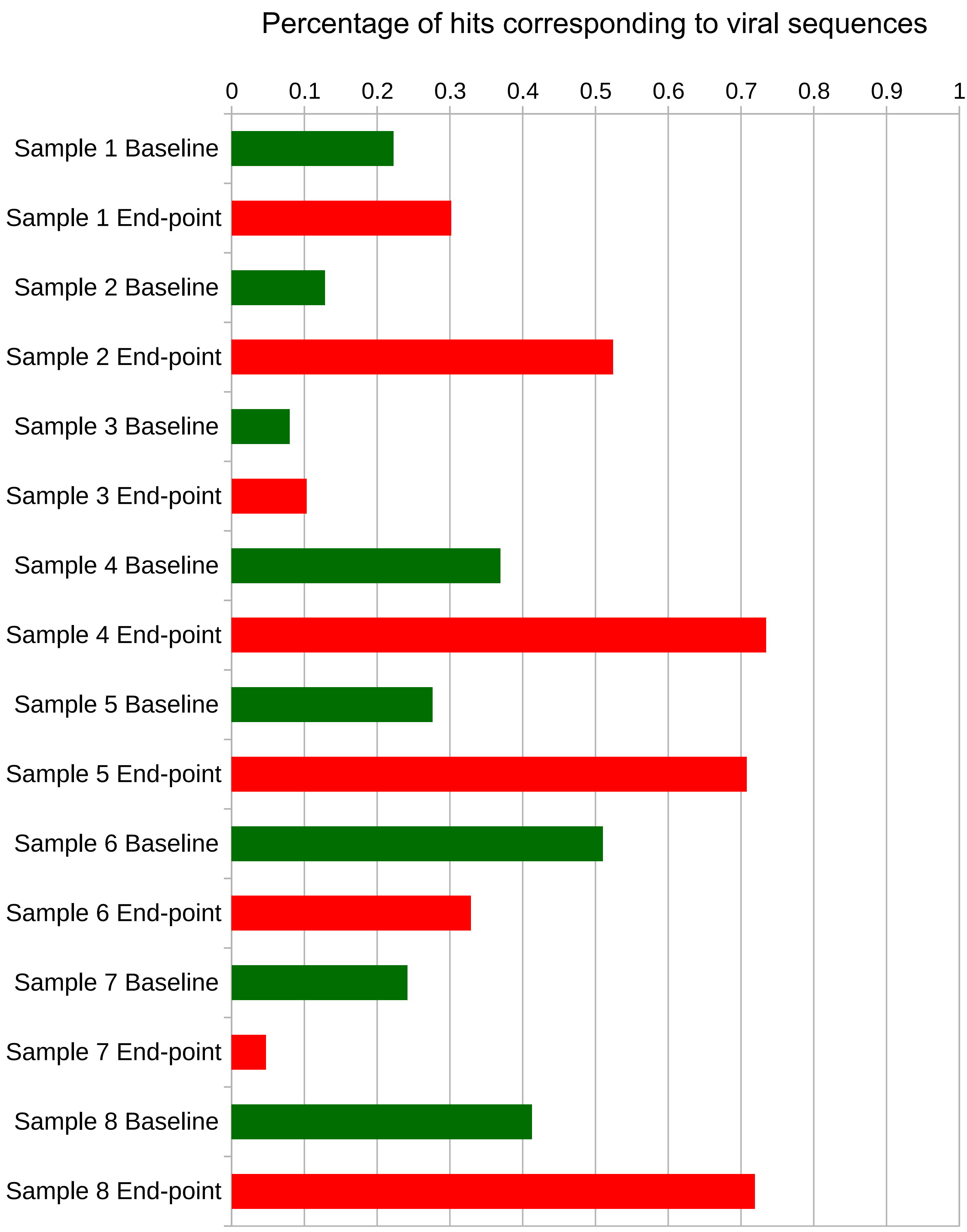

Figure S13. Percentage of hits corresponding to viral sequences. Sequences were aligned against a database containing all viral sequences in NCBI. Bars represent the percentage of hits that corresponded to viral sequences.

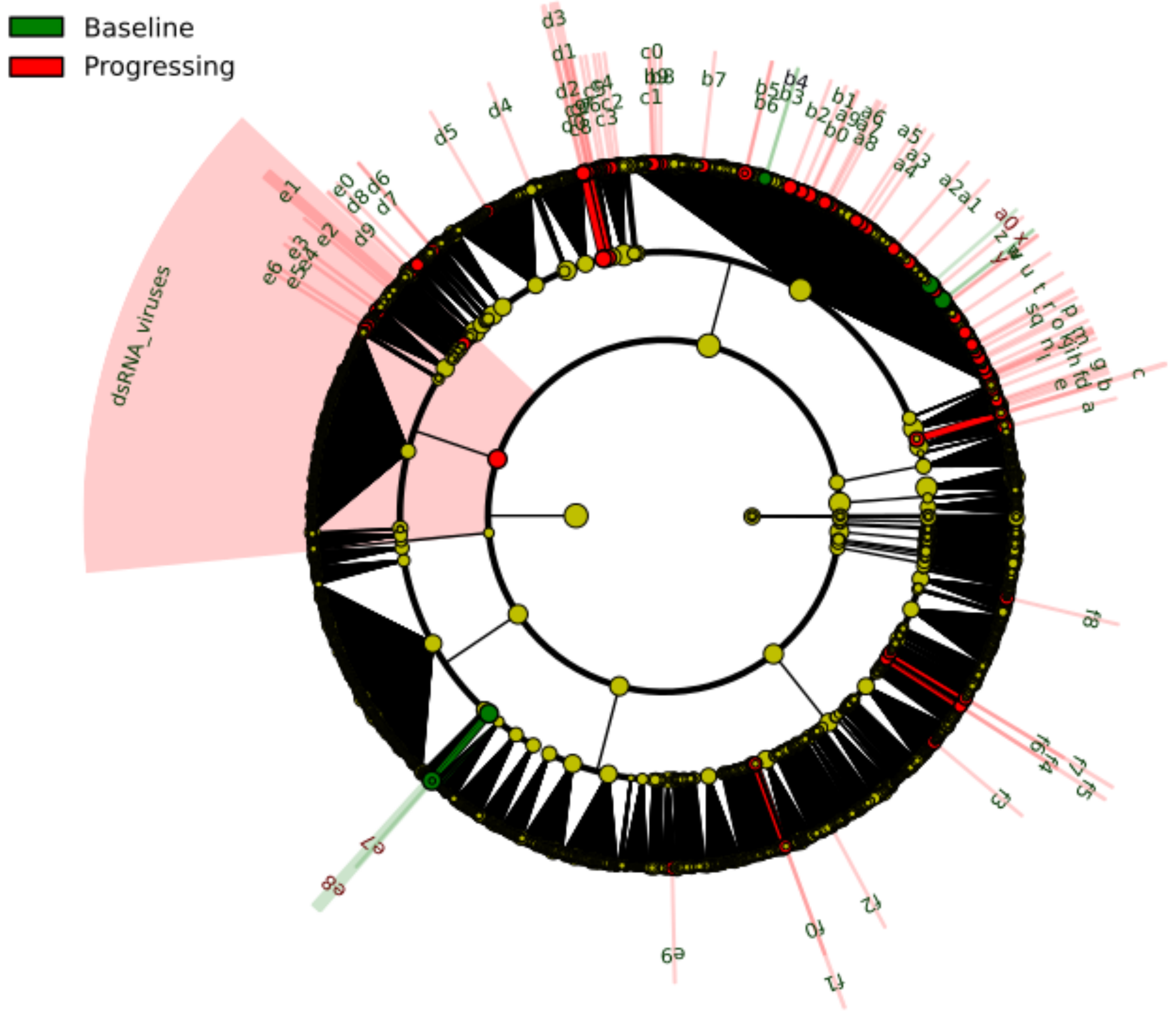

- a: Fowl_adenovirus_C
- b: Heliothis_virescens_ascovirus_3e
- c: Ascoviridae
- d: Bombyx_mori_NPV
- e: Chrysodeixis_chalcites_nucleopolyhedrovirus
- f: Epinotia_aporema_granulovirus
- g: Erwinia_phage_ENT90
- h: Rachiplusia_ou_MNPV
- i: Acinetobacter_phage_Acj9
- j: Aeromonas_phage_31
- k: Aeromonas_phage_Aes012
- l: Aeromonas_phage_phiAS4
- m: Agrobacterium_phage_7_7_1
- n: Bacillus_phage_BPS13
- o: Bacillus_phage_Megatron
- p: Bacillus_phage_SPBc2
- q: Bacteriophage_RB32
- r: Burkholderia_phage_AH2
- s: Burkholderia_phage_BcepB1A
- t: Cellulophaga_phage_phi14_2
- u: Cronobacter_phage_ENT39118
- v: Enterobacteria_phage_CC31
- w: Enterobacteria_phage_EPS7
- z: Enterobacteria_phage_RTP
- x: Enterobacteria_phage_HK629
- y: Enterobacteria_phage_HK630
- a0: Enterobacteria_phage_lambda
- b4: Pseudomonas_phage_MP38
- e7: Enterobacteria_phage_phiX174_sensu_lato
- e8: Microviridae
- a1: Haemophilus_phage_HP2
- a2: Lactococcus_phage_P092
- a3: Mycobacterium_phage_Artemis2UCLA
- a4: Mycobacterium_phage_Bruin
- a5: Mycobacterium_phage_Che8
- a6: Mycobacterium_phage_MichelleMyBell
- a7: Mycobacterium_phage_Obama12
- a8: Mycobacterium_phage_PLot
- a9: Mycobacterium_phage_Spud
- b0: Mycobacterium_phage_Thibault
- b1: Myobacteriophage_Papyrus
- b2: Prochlorococcus_phage_P_SSM2
- b3: Pseudomonas_phage_MP29
- b5: Ralstonia_phage_RSB3_DNA
- b6: Ralstonia_phage_RSL1
- b7: Staphylococcus_phage_EW
- b8: Synechococcus_phage_S_IOM18
- b9: Synechococcus_phage_S_SSM5
- c0: Thermus_phage_P23_45
- c1: Thermus_phage_TMA
- e9: Mouse_astrovirus_M_52_USA_2008
- f0: Fathead_minnow_picornavirus_strain_09_283
- f1: Fathead_minnow_picornavirus
- f2: Bat_hepevirus
- f3: Papaya_ringspot_virus
- f4: Seneca_valley_virus
- f5: Seneca_valley_virus
- f6: Solenopsis_invicta_virus_3
- f7: Solenopsis_invicta_virus_1
- f8: Pepper_mild_mottle_virus
- c2: Callitrichine_herpesvirus_3_strain_CJ0149
- c3: Equid_herpesvirus_1
- c4: Gallid_herpesvirus_2
- c5: Meleagrid_herpesvirus_1
- c6: Ranid_herpesvirus_1_strain_McKinnell
- c7: Invertebrate_iridovirus_25_complete
- c8: Lymphocystis_disease_virus___isolate_China
- c9: Acidianus_filamentous_virus_9
- d0: Sulfolobus_islandicus_rod_shaped_virus_1
- d1: Ligamenvirales
- d2: Helicoverpa_zea_nudivirus_2
- d3: Nudiviridae
- d4: Phaeocystis_globosa_virus_strain_16T
- d5: Glypta_fumiferanae_ichnovirus_segment_C10
- d6: Pigeonpox_virus_isolate_FeP2
- d7: Rabbit_fibroma_virus
- d8: Vibrio_phage_henriette_12B8
- d9: Halovirus_PH1
- e0: Micromonas_pusilla_virus_12T
- e1: Chrysoviridae
- e2: Pseudomonas_phage_phi15
- e3: Sclerotinia_sclerotiorum_hypovirus_2_isolate_5472
- e4: Dill_cryptic_virus_1_isolate_IPP_hortorum_segment_1
- e5: Rhizoctonia_solani_dsRNA_virus_2_segment_2
- e6: Vicia_cryptic_virus_RNA2

Figure S14. Statistical differences in viral composition of transcript libraries. Hit counts were analyzed using LEfSe with default parameters, to identify significant differences at species level between the microbial communities compared. Comparison progressing site baselines to end point of the same sites.
