# Supplementary material for: Functional signatures of oral dysbiosis during periodontitis progression revealed by microbial metatranscriptome analysis
Source: Genome Med. 2015 Apr 27;7(1):27. doi: 10.1186/s13073-015-0153-3 (PMC4410737; doi:10.1186/s13073-015-0153-3)
Supplement: Additional file 6: Table S4. — Differentially expressed (DE) putative virulence factors of the red complex when comparing the baseline expression profiles of active and inactive sites. Based on the VFDB (see Methods) we compared expression of DE putative virulence factors at baseline and selected those corresponding to the red complex. [file 13073_2015_153_MOESM6_ESM.doc]

### Table S4. **Differentially expressed (DE) putative virulence factors of the red complex when comparing the baseline expression profiles of active and inactive sites.** Based on the VFDB (see Material and Methods) we compared expression of DE putative virulence factors at baseline and selected those corresponding to the red complex.

|  | |
| --- | --- |
| **Up-regulated putative virulent factors** | |
| VBIPorGin134034_0117 | FIG00936601_hypothetical_protein |
| VBIPorGin134034_0126 | Malonyl_CoA-acyl_carrier_protein_transacylase_(EC_2.3.1.39) |
| VBIPorGin134034_0661 | DNA-binding_response_regulator |
| VBIPorGin134034_1006 | Transcriptional_regulatory_protein_rprY |
| VBIPorGin134034_1449 | Glucose-1-phosphate_thymidylyltransferase_(EC_2.7.7.24) |
| VBIPorGin195531_0043 | Diaminopimelate_decarboxylase_(EC_4.1.1.20) |
| VBIPorGin195531_0328 | N-acetylglucosamine_deacetylase_(EC_3.5.1.-)_(3R)-hydroxymyristoyl-acyl_carrier_protein_dehydratase_(EC_4.2.1.-) |
| VBIPorGin195531_0343 | Arabinose_5-phosphate_isomerase_(EC_5.3.1.13) |
| VBIPorGin195531_0728 | Manganese_superoxide_dismutase_(EC_1.15.1.1) |
| VBIPorGin195531_0929 | Ferrous_iron_transport_protein_B |
| VBIPorGin239392_0232 | FKBP-type_peptidyl-prolyl_cis-trans_isomerase_FkpA_precursor_(EC_5.2.1.8) |
| VBIPorGin239392_0348 | Aspartate_1-decarboxylase_(EC_4.1.1.11) |
| VBIPorGin239392_0409 | oxidoreductase_Gfo_Idh_MocA_family |
| VBIPorGin239392_0414 | NAD-dependent_glyceraldehyde-3-phosphate_dehydrogenase_(EC_1.2.1.12) |
| VBIPorGin239392_0444 | 3-oxoacyl-acyl-carrier-proteinsynthase_KASII_(EC_2.3.1.41) |
| VBIPorGin239392_0543 | Translation_elongation_factor_Tu |
| VBIPorGin239392_0764 | UDP-3-O-3-hydroxymyristoyl_glucosamine_N-acyltransferase_(EC_2.3.1.-) |
| VBIPorGin239392_1219 | ABC_transporter_ATP-binding_protein |
| VBIPorGin239392_1345 | Lipopolysaccharide_ABC_transporter_ATP-binding_protein_LptB |
| VBIPorGin239392_1442 | dTDP-4-dehydrorhamnose_reductase_(EC_1.1.1.133) |
| VBIPorGin239392_1690 | HtrA_protease_chaperone_protein |
| VBIPorGin239392_1854 | ABC_transporter_ATP-binding_protein_YvcR |
| VBIPorGin239392_1870 | Tyrosine-protein_kinase_Wzc_(EC_2.7.10.2) |
| VBIPorGin239392_1916 | Calcium-transporting_ATPase |
| VBIPorGin239392_1926 | UDP-N-acetyl-D-mannosamine_dehydrogenase_(EC_1.1.1.-) |
| VBIPorGin26334_0090 | Cell_division_transporter_ATP-binding_protein_FtsE_(TC_3.A.5.1.1) |
| VBIPorGin26334_0543 | dTDP-4-dehydrorhamnose_3_5-epimerase_(EC_5.1.3.13) |
| VBIPorGin26334_0556 | Manganese_superoxide_dismutase_(EC_1.15.1.1) |
| VBIPorGin26334_0651 | Alkyl_hydroperoxide_reductase_protein_C_(EC_1.6.4.-) |
| VBIPorGin26334_0742 | DNA-binding_response_regulator |
| VBIPorGin26334_1179 | Transcriptional_regulatory_protein_rprY |
| VBIPorGin26334_1559 | Translation_elongation_factor_Tu |
| VBIPorGin26334_1719 | Enolase_(EC_4.2.1.11) |
| VBIPorGin26334_1769 | leucine-rich_protein |
| VBITanFor42681_1265 | putative_bactoprenol_glucosyl_transferase-like_protein |
| VBITreDen445_0573 | internalin-related_protein |
| VBITreDen445_0897 | Ornithine_carbamoyltransferase_(EC_2.1.3.3) |
| VBITreDen445_2483 | Transport_ATP-binding_protein_CydD |
| **Down-regulated putative virulent factors** | |
| VBIPorGin134034_0354 | Translation_elongation_factor_Tu |
| VBIPorGin134034_1631 | 3-oxoacyl-acyl-carrier-proteinsynthase_KASII_(EC_2.3.1.41) |
| VBIPorGin26334_0168 | NAD-dependent_glyceraldehyde-3-phosphate_dehydrogenase_(EC_1.2.1.12) |
| VBIPorGin26334_0729 | FKBP-type_peptidyl-prolyl_cis-trans_isomerase_FkpA_precursor_(EC_5.2.1.8) |
| VBIPorGin26334_0939 | ABC_transporter_ATP-binding_protein |
| VBIPorGin26334_1517 | Carbonic_anhydrase_(EC_4.2.1.1) |
| VBIPorGin26334_1888 | ABC_transporter_ATP-binding_protein |
