# Supplementary material for: Functional signatures of oral dysbiosis during periodontitis progression revealed by microbial metatranscriptome analysis
Source: Genome Med. 2015 Apr 27;7(1):27. doi: 10.1186/s13073-015-0153-3 (PMC4410737; doi:10.1186/s13073-015-0153-3)
Supplement: Additional file 7: Table S5. — Differentially expressed (DE) putative virulence factors of the orange complex when comparing the baseline expression profiles of active and inactive sites. Based on the VFDB (see Methods) we compared expression of DE putative virulence factors at baseline and selected those corresponding to the orange complex. [file 13073_2015_153_MOESM7_ESM.doc]

### **Table S5. Differentially expressed (DE) putative virulence factors of the orange complex when comparing the baseline expression profiles of active and inactive sites.** Based on the VFDB (see Material and Methods) we compared expression of DE putative virulence factors at baseline and selected those corresponding to the orange complex.

| **Up-regulated putative virulent factors** | |
| --- | --- |
| **Locus** | **Function** |
| VBICamGra90913_0502 | Acyl-acyl-carrier-protein--UDP-N-acetylglucosamine_O-acyltransferase_(EC_2.3.1.129) |
| VBICamGra90913_1696 | FOG_TPR_repeat |
| VBICamGra90913_1696 | FOG_TPR_repeat |
| VBICamGra90913_1696 | FOG_TPR_repeat |
| VBICamGra90913_1696 | FOG_TPR_repeat |
| VBICamGra90913_1696 | FOG_TPR_repeat |
| VBICamGra90913_1696 | FOG_TPR_repeat |
| VBICamGra90913_1696 | FOG_TPR_repeat |
| VBICamGra90913_1736 | Rrf2_family_transcriptional_regulator |
| VBICamGra90913_1777 | Acetylornithine_aminotransferase_(EC_2.6.1.11) |
| VBICamGra90913_2195 | Translation_elongation_factor_Tu |
| VBICamGra90913_2265 | ABC_transporter_ATP-binding_protein |
| VBICamGra90913_2435 | Arabinose_5-phosphate_isomerase_(EC_5.3.1.13) |
| VBICamGra90913_2507 | Possible_bacterioferritin |
| VBICamGra90913_2565 | Putative_two-domain_glycosyltransferase |
| VBICamGra90913_2591 | Leader_peptidase_(Prepilin_peptidase)_(EC_3.4.23.43)_N-methyltransferase_(EC_2.1.1.-) |
| VBICamGra90913_2641 | UDP-N-acetylglucosamine_4_6-dehydratase_(EC_4.2.1.-) |
| VBICamGra90913_2650 | Alpha-1_4-N-acetylgalactosamine_transferase_PglH_(EC_2.4.1.-) |
| VBICamGra90913_2700 | 3-oxoacyl-acyl-carrier-proteinsynthase_KASII_(EC_2.3.1.41) |
| VBICamRec32822_0025 | Vitamin_B12_ABC_transporter_permease_component_BtuC |
| VBICamRec32822_0577 | Translation_elongation_factor_Tu |
| VBICamRec32822_0879 | Zinc_ABC_transporter_ATP-binding_protein_ZnuC |
| VBICamRec32822_1086 | Iron(III)_ABC_transporter_ATP-binding_protein |
| VBICamRec32822_1520 | Superoxide_dismutase_Cu-Zn_precursor_(EC_1.15.1.1) |
| VBIFusNuc113593_0961 | Heat_shock_protein_60_family_chaperone_GroEL |
| VBIFusNuc72376_0156 | Dipeptide_transport_ATP-binding_protein_DppD_(TC_3.A.1.5.2) |
| VBIFusNuc72376_0272 | Translation_elongation_factor_Tu |
| VBIFusNuc72376_0945 | Lipopolysaccharide_ABC_transporter_ATP-binding_protein_LptB |
| VBIFusNuc72376_1314 | Porphobilinogen_synthase_(EC_4.2.1.24) |
| VBIFusNuc72376_1615 | ABC_transporter_ATP-binding_protein |
| VBIFusPer130431_0813 | Lipid_carrier_UDP-N-acetylgalactosaminyltransferase_(EC_2.4.1.-) |
| VBIParMic42033_0050 | Translation_elongation_factor_Tu |
| VBIParMic42033_0271 | ESAT-6_Esx_family_secreted_protein_EsxA_YukE |
| VBIParMic42033_0432 | Catabolite_control_protein_A |
| VBIParMic42033_0527 | hypothetical_protein |
| VBIParMic42033_0600 | Oligopeptide_transport_ATP-binding_protein_OppD_(TC_3.A.1.5.1) |
| VBIParMic42033_0601 | Oligopeptide_transport_ATP-binding_protein_OppF_(TC_3.A.1.5.1) |
| VBIParMic42033_0609 | UDP-4-amino-4-deoxy-L-arabinose--oxoglutarate_aminotransferase_(EC_2.6.1.-) |
| VBIParMic42033_0624 | 2-amino-3-ketobutyrate_coenzyme_A_ligase_(EC_2.3.1.29) |
| VBIParMic42033_0625 | Serine_protease_DegP_HtrA_do-like_(EC_3.4.21.-) |
| VBIParMic42033_0837 | V-type_ATP_synthase_subunit_A_(EC_3.6.3.14) |
| VBIParMic42033_1254 | Hemolysins_and_related_proteins_containing_CBS_domains |
| VBIParMic42033_1277 | Alkyl_hydroperoxide_reductase_protein_C_(EC_1.6.4.-) |
| VBIParMic42033_1412 | NAD-dependent_glyceraldehyde-3-phosphate_dehydrogenase_(EC_1.2.1.12) |
| VBIParMic42033_1439 | NAD-dependent_formate_dehydrogenase_alpha_subunit_selenocysteine-containing |
| VBIPreInt119176_0010 | TonB-dependent_receptor |
| VBIPreInt119176_0503 | Cell_division_transporter_ATP-binding_protein_FtsE_(TC_3.A.5.1.1) |
| VBIPreInt119176_0601 | Phosphatidate_cytidylyltransferase_(EC_2.7.7.41) |
| VBIPreInt119176_0632 | Methionine_ABC_transporter_ATP-binding_protein |
| VBIPreInt119176_0634 | Lipopolysaccharide_ABC_transporter_ATP-binding_protein_LptB |
| VBIPreInt119176_0667 | GTP_pyrophosphokinase_(EC_2.7.6.5)_(p)ppGpp_synthetase_II_Guanosine-3'_5'-bis(diphosphate)_3'-pyrophosphohydrolase_(EC_3.1.7.2) |
| VBIPreInt119176_0680 | DNA-binding_response_regulator_AraC_family |
| VBIPreInt119176_0730 | Lipid_A_export_ATP-binding_permease_protein_MsbA |
| VBIPreInt119176_0848 | Calcium-transporting_ATPase |
| VBIPreInt119176_0879 | streptococcal_pyrogenic_exotoxin_B(_EC_3.4.22.10_) |
| VBIPreInt119176_1011 | 3-oxoacyl-acyl-carrier-proteinsynthase_KASII_(EC_2.3.1.41) |
| VBIPreInt119176_1060 | FKBP-type_peptidyl-prolyl_cis-trans_isomerase_fklB_(EC_5.2.1.8) |
| VBIPreInt119176_1061 | FKBP-type_peptidyl-prolyl_cis-trans_isomerase_fklB_(EC_5.2.1.8) |
| VBIPreInt119176_1062 | FKBP-type_peptidyl-prolyl_cis-trans_isomerase_fklB_(EC_5.2.1.8) |
| VBIPreInt119176_1124 | ABC_transporter_ATP-binding_protein |
| VBIPreInt119176_1128 | Ferric_enterobactin_transport_ATP-binding_protein_FepC_(TC_3.A.1.14.2) |
| VBIPreInt119176_1160 | Mannose-1-phosphate_guanylyltransferase_(GDP)_(EC_2.7.7.22) |
| VBIPreInt119176_1402 | Translation_elongation_factor_Tu |
| VBIPreInt119176_1414 | Internalin-like_protein_(LPXTG_motif)_Lmo0331_homolog |
| VBIPreInt119176_1487 | ABC_transporter_ATP-binding_permease_protein |
| VBIPreInt119176_1487 | ABC_transporter_ATP-binding_permease_protein |
| VBIPreInt119176_1529 | protein_of_unknown_function_DUF323 |
| VBIPreInt119176_1629 | UDP-glucose_4-epimerase_(EC_5.1.3.2) |
| VBIPreInt119176_1660 | ABC_transporter_ATP-binding_protein_putative |
| VBIPreInt119176_1684 | Mannose-6-phosphate_isomerase_(EC_5.3.1.8) |
| VBIPreInt119176_1692 | Phosphomannomutase_(EC_5.4.2.8) |
| VBIPreInt119176_1702 | UDP-glucose_dehydrogenase_(EC_1.1.1.22) |
| VBIPreInt119176_1716 | Malonyl_CoA-acyl_carrier_protein_transacylase_(EC_2.3.1.39) |
| VBIPreInt119176_1727 | hypothetical_protein |
| VBIPreInt119176_1738 | NAD-dependent_glyceraldehyde-3-phosphate_dehydrogenase_(EC_1.2.1.12) |
| VBIPreInt119176_1745 | Transcriptional_regulator |
| VBIPreInt119176_1760 | Glycosyl_transferase |
| VBIPreInt119176_1884 | GDP-mannose_4_6_dehydratase_(EC_4.2.1.47) |
| VBIPreInt119176_1927 | 8-amino-7-oxononanoate_synthase_(EC_2.3.1.47) |
| VBIPreInt119176_1986 | GDP-L-fucose_synthetase_(EC_1.1.1.271) |
| VBIPreInt119176_2006 | UDP-N-acetylglucosamine_4_6-dehydratase_(EC_4.2.1.-) |
| VBIPreInt119176_2040 | Hemolysins_and_related_proteins_containing_CBS_domains |
| VBIPreInt119176_2106 | Heat_shock_protein_60_family_chaperone_GroEL |
| VBIPreInt119176_2124 | dTDP-4-dehydrorhamnose_reductase_(EC_1.1.1.133) |
| VBIPreInt119176_2151 | ATP_synthase_alpha_chain_(EC_3.6.3.14) |
| VBIPreInt119176_2158 | ATP_synthase_beta_chain_(EC_3.6.3.14) |
| VBIPreInt119176_2164 | glycosyltransferase |
| VBIPreInt119176_2194 | dTDP-glucose_4_6-dehydratase_(EC_4.2.1.46) |
| VBIPreInt119176_2197 | Aminotransferase |
| VBIPreInt119176_2220 | capsular_polysaccharide_synthesis_enzyme_Cap8H |
| VBIPreInt119176_2232 | Alkyl_hydroperoxide_reductase_protein_C_(EC_1.6.4.-) |
| VBIPreInt119176_2248 | Arabinose_5-phosphate_isomerase_(EC_5.3.1.13) |
| VBIPreInt119176_2264 | Tyrosine-protein_kinase_Wzc_(EC_2.7.10.2) |
| VBIPreInt119176_2275 | UDP-N-acetylglucosamine_2-epimerase_(EC_5.1.3.14) |
| VBIPreInt119176_2297 | ClpB_protein |
| VBIPreInt119176_2335 | Probable_poly(beta-D-mannuronate)_O-acetylase_(EC_2.3.1.-) |
| VBIPreInt119176_2366 | Acyl-acyl-carrier-protein--UDP-N-acetylglucosamine_O-acyltransferase_(EC_2.3.1.129) |
| VBIPreInt119176_2407 | internalin-related_protein |
| VBIPreInt119176_2418 | Outer_membrane_stress_sensor_protease_DegS |
| VBIPreInt119176_2447 | lipoprotein_releasing_system_ATP-binding_protein |
| VBIPreNig185369_0834 | Glycosyl_transferase |
| VBIPreNig185369_1217 | FKBP-type_peptidyl-prolyl_cis-trans_isomerase_fklB_(EC_5.2.1.8) |
| VBIPreNig185369_1218 | FKBP-type_peptidyl-prolyl_cis-trans_isomerase_fklB_(EC_5.2.1.8) |
| VBIPreNig185369_1305 | Ferric_enterobactin_transport_ATP-binding_protein_FepC_(TC_3.A.1.14.2) |
| VBIPreNig185369_1841 | hypothetical_protein |
| VBIPreNig185369_2187 | Lipoate_synthase |
| VBIPreNig185369_2234 | streptopain |
| VBIStrCon222224_0062 | Enolase_(EC_4.2.1.11) |
| VBIStrCon222224_0076 | Hemolysin_III |
| VBIStrCon222224_0091 | Two_component_system_response_regulator_CiaR |
| VBIStrCon222224_0115 | Glutamate_transport_ATP-binding_protein |
| VBIStrCon222224_0200 | Response_regulator_CsrR |
| VBIStrCon222224_0223 | Sortase_A_LPXTG_specific |
| VBIStrCon222224_0246 | Acetoin_dehydrogenase_E1_component_beta-subunit_(EC_1.2.4.-) |
| VBIStrCon222224_0249 | Lipoate-protein_ligase_A |
| VBIStrCon222224_0280 | RNA_polymerase_sigma_factor_RpoD |
| VBIStrCon222224_0453 | Translation_elongation_factor_Tu |
| VBIStrCon222224_0502 | L-proline_glycine_betaine_ABC_transport_system_permease_protein_ProV_(TC_3.A.1.12.1) |
| VBIStrCon222224_0628 | ATP-dependent_Clp_protease_ATP-binding_subunit_ClpE |
| VBIStrCon222224_0707 | Mg(2+)_transport_ATPase_P-type_(EC_3.6.3.2) |
| VBIStrCon222224_0730 | Histidine_kinase |
| VBIStrCon222224_0749 | Hemolysins_and_related_proteins_containing_CBS_domains |
| VBIStrCon222224_0793 | Putrescine_transport_ATP-binding_protein_PotA_(TC_3.A.1.11.1) |
| VBIStrCon222224_0816 | Cell_division_transporter_ATP-binding_protein_FtsE_(TC_3.A.5.1.1) |
| VBIStrCon222224_0859 | ATP_synthase_alpha_chain_(EC_3.6.3.14) |
| VBIStrCon222224_0861 | ATP_synthase_beta_chain_(EC_3.6.3.14) |
| VBIStrCon222224_0866 | ABC_transporter_ATP-binding_permease_protein |
| VBIStrCon222224_0897 | NAD-dependent_glyceraldehyde-3-phosphate_dehydrogenase_(EC_1.2.1.12) |
| VBIStrCon222224_0899 | Heat_shock_protein_60_family_chaperone_GroEL |
| VBIStrCon222224_0921 | Phosphatidate_cytidylyltransferase_(EC_2.7.7.41) |
| VBIStrCon222224_0922 | Undecaprenyl_pyrophosphate_synthetase_(EC_2.5.1.31)_Di-trans_poly-cis-decaprenylcistransferase |
| VBIStrCon222224_0934 | putative_ATP-dependent_Clp_proteinase_(ATP-binding_subunit) |
| VBIStrCon222224_0934 | putative_ATP-dependent_Clp_proteinase_(ATP-binding_subunit) |
| VBIStrCon222224_0946 | Glutamine_synthetase_type_I_(EC_6.3.1.2) |
| VBIStrCon222224_0983 | GTP_pyrophosphokinase_(EC_2.7.6.5)_(p)ppGpp_synthetase_I |
| VBIStrCon222224_0986 | Manganese_ABC_transporter_periplasmic-binding_protein_SitA |
| VBIStrCon222224_0987 | Manganese_ABC_transporter_inner_membrane_permease_protein_SitD |
| VBIStrCon222224_0988 | Manganese_ABC_transporter_ATP-binding_protein_SitB |
| VBIStrCon222224_0997 | Serine_threonine_protein_kinase_PrkC_regulator_of_stationary_phase |
| VBIStrCon222224_0998 | Protein_serine_threonine_phosphatase_PrpC_regulation_of_stationary_phase |
| VBIStrCon222224_1005 | S-ribosylhomocysteine_lyase_(EC_4.4.1.21)_Autoinducer-2_production_protein_LuxS |
| VBIStrCon222224_1007 | Alkyl_hydroperoxide_reductase_protein_C_(EC_1.6.4.-) |
| VBIStrCon222224_1020 | ABC-type_multidrug_transport_system_ATPase_component |
| VBIStrCon222224_1028 | Undecaprenyl-phosphate_N-acetylglucosaminyl_1-phosphate_transferase_(EC_2.7.8.-) |
| VBIStrCon222224_1033 | Amino_acid_transport_ATP-binding_protein |
| VBIStrCon222224_1085 | Calcium-transporting_ATPase_(EC_3.6.3.8) |
| VBIStrCon222224_1111 | Ornithine_carbamoyltransferase_(EC_2.1.3.3) |
| VBIStrCon222224_1127 | Manganese_superoxide_dismutase_(EC_1.15.1.1) |
| VBIStrCon222224_1140 | Peptidyl-prolyl_cis-trans_isomerase_(EC_5.2.1.8) |
| VBIStrCon222224_1214 | Protein_export_cytoplasm_protein_SecA_ATPase_RNA_helicase_(TC_3.A.5.1.1) |
| VBIStrCon222224_1216 | Mannose-6-phosphate_isomerase_(EC_5.3.1.8) |
| VBIStrCon222224_1222 | Lipid_A_export_ATP-binding_permease_protein_MsbA |
| VBIStrCon222224_1223 | Lipid_A_export_ATP-binding_permease_protein_MsbA |
| VBIStrCon222224_1233 | Oligopeptide_transport_ATP-binding_protein_OppF_(TC_3.A.1.5.1) |
| VBIStrCon222224_1234 | Oligopeptide_transport_ATP-binding_protein_OppD_(TC_3.A.1.5.1) |
| VBIStrCon222224_1238 | Oligopeptide_ABC_transporter_periplasmic_oligopeptide-binding_protein_OppA_(TC_3.A.1.5.1) |
| VBIStrCon222224_1243 | Cell_division_trigger_factor_(EC_5.2.1.8) |
| VBIStrCon222224_1274 | cell_wall_surface_anchor_family_protein |
| VBIStrCon222224_1280 | UDP-N-acetylglucosamine_2-epimerase_(EC_5.1.3.14) |
| VBIStrCon222224_1296 | Multiple_sugar_ABC_transporter_ATP-binding_protein |
| VBIStrCon222224_1338 | Serine_protease_DegP_HtrA_do-like_(EC_3.4.21.-) |
| VBIStrCon222224_1356 | ATPase_component_of_general_energizing_module_of_ECF_transporters |
| VBIStrCon222224_1357 | ATPase_component_of_general_energizing_module_of_ECF_transporters |
| VBIStrCon222224_1387 | D-alanine--poly(phosphoribitol)_ligase_subunit_1_(EC_6.1.1.13) |
| VBIStrCon222224_1388 | D-alanyl_transfer_protein_DltB |
| VBIStrCon222224_1422 | Alcohol_dehydrogenase_(EC_1.1.1.1)_Acetaldehyde_dehydrogenase_(EC_1.2.1.10) |
| VBIStrCon222224_1439 | V-type_ATP_synthase_subunit_A_(EC_3.6.3.14) |
| VBIStrCon222224_1456 | Transport_ATP-binding_protein_CydC |
| VBIStrCon222224_1537 | Two-component_sensor_kinase_SA14-24 |
| VBIStrCon222224_1538 | Two-component_response_regulator_SA14-24 |
| VBIStrCon222224_1541 | Choline_kinase_(EC_2.7.1.32) |
| VBIStrCon222224_1548 | Lipopolysaccharide_cholinephosphotransferase_LicD1_(EC_2.7.8.-) |
| VBIStrCon222224_1549 | putative_glycosyl_transferase |
| VBIStrCon222224_1555 | Undecaprenyl-phosphate_galactosephosphotransferase_(EC_2.7.8.6) |
| VBIStrCon222224_1557 | Tyrosine-protein_kinase_transmembrane_modulator_EpsC |
| VBIStrCon222224_1558 | Tyrosine-protein_phosphatase_CpsB_(EC_3.1.3.48) |
| VBIStrCon222224_1559 | Exopolysaccharide_biosynthesis_transcriptional_activator_EpsA |
| VBIStrCon222224_1581 | putative_phosphomannomutase |
| VBIStrCon222224_1613 | ABC-type_multidrug_transport_system_ATPase_component |
| VBIStrCon222224_1624 | Transport_ATP-binding_protein_CydD |
| VBIStrCon222224_1625 | Transport_ATP-binding_protein_CydC |
| VBIStrCon222224_1641 | Phosphate_transport_ATP-binding_protein_PstB_(TC_3.A.1.7.1) |
| VBIStrCon222224_1656 | Phosphoglucomutase_(EC_5.4.2.2) |
| VBIStrCon222224_1683 | UTP--glucose-1-phosphate_uridylyltransferase_(EC_2.7.7.9) |
| VBIStrCon222224_1725 | C3-degrading_proteinase |
| VBIStrCon222224_1748 | Phosphoglucosamine_mutase_(EC_5.4.2.10) |
| VBIStrCon222224_1766 | Prolipoprotein_diacylglyceryl_transferase_(EC_2.4.99.-) |
| VBIStrCon222224_1784 | Two-component_sensor_kinase_SA14-24 |
| VBIStrCon222224_1785 | Two-component_response_regulator_SA14-24 |
| VBIStrCon222224_1789 | Catabolite_control_protein_A |
| VBIStrCon222224_1792 | UDP-glucose_4-epimerase_(EC_5.1.3.2) |
| VBIStrCon222224_1793 | dTDP-glucose_4_6-dehydratase_(EC_4.2.1.46) |
| VBIStrCon222224_1794 | dTDP-4-dehydrorhamnose_3_5-epimerase_(EC_5.1.3.13) |
| VBIStrCon222224_1795 | Glucose-1-phosphate_thymidylyltransferase_(EC_2.7.7.24) |
| VBIStrCon222224_1801 | Alpha-L-Rha_alpha-1_2-L-rhamnosyltransferase_alpha-L-Rha_alpha-1_3-L-_rhamnosyltransferase_(EC_2.4.1.-) |
| VBIStrCon222224_1802 | ABC-transporter_(ATP-binding_protein)_-possibly_involved_in_cell_wall_localization_and_side_chain_formation_of_rhamnose-glucose_polysaccharide |
| VBIStrCon222224_1803 | rhamnose-containing_polysacharide_translocation_permease |
| VBIStrCon222224_1804 | Alpha-L-Rha_alpha-1_3-L-rhamnosyltransferase_(EC_2.4.1.-) |
| VBIStrCon222224_1805 | Alpha-D-GlcNAc_alpha-1_2-L-rhamnosyltransferase_(EC_2.4.1.-) |
| VBIStrCon222224_1806 | glycosyltransferase |
| VBIStrCon222224_1807 | membrane_protein_related_to_Actinobacillus_protein_(1944168) |
| VBIStrCon222224_1808 | Lipopolysaccharide_biosynthesis_protein |
| VBIStrCon222224_1809 | Glycosyl_transferase_family_2 |
| VBIStrCon222224_1810 | DNA_for_glycosyltransferase_lytic_transglycosylase_dTDP-4-rhamnose_reductase |
| VBIStrCon222224_1811 | Glycosyltransferases_involved_in_cell_wall_biogenesis |
| VBIStrCon222224_1812 | Beta-1_3-glucosyltransferase |
| VBIStrCon222224_1813 | Putative_CDP-glycosylpolyol_phosphate_glycosylpolyol_glycosylpolyolphosphotransferase |
| VBIStrCon222224_1814 | Heteropolysaccharide_repeat_unit_export_protein |
| VBIStrCon222224_1815 | dTDP-4-dehydrorhamnose_reductase_(EC_1.1.1.133) |
| VBIStrCon222224_1817 | Bactoprenol_glucosyl_transferase_(EC_2.4.1.-) |
|  |  |
| **Down-regulated putative virulent factors** | |
| **Locus** | **Function** |
| VBICamRec32822_0575 | HlyD_family_secretion_protein |
| VBICamRec32822_1032 | UTP--glucose-1-phosphate_uridylyltransferase_(EC_2.7.7.9) |
| VBICamRec32822_2462 | Putative_ABC_transport_system_ATP-binding_protein |
| VBICamSho45867_1475 | C4_aminotransferase_specific_for_PseB_product_(PseC_second_step_of_pseudaminic_acid_biosynthesis) |
| VBIFusNuc113593_0982 | Lipopolysaccharide_ABC_transporter_ATP-binding_protein_LptB |
| VBIFusNuc113593_1966 | Type_IV_fimbrial_assembly_ATPase_PilB |
| VBIFusNuc247698_0166 | ATP_synthase_beta_chain_(EC_3.6.3.14) |
| VBIFusNuc247698_0168 | ATP_synthase_alpha_chain_(EC_3.6.3.14) |
| VBIFusNuc247698_0195 | (3R)-hydroxymyristoyl-acyl_carrier_protein_dehydratase_(EC_4.2.1.-) |
| VBIFusNuc247698_0310 | Oligopeptide_transport_ATP-binding_protein_OppD_(TC_3.A.1.5.1) |
| VBIFusNuc247698_0390 | Protein_export_cytoplasm_protein_SecA_ATPase_RNA_helicase_(TC_3.A.5.1.1) |
| VBIFusNuc247698_0686 | Translation_elongation_factor_Tu |
| VBIParMic42033_0057 | Ornithine_carbamoyltransferase_(EC_2.1.3.3) |
| VBIStrCon222224_0994 | Two_component_transcriptional_regulator_VraR |
| VBIStrCon222224_1440 | V-type_ATP_synthase_subunit_B_(EC_3.6.3.14) |
|  |  |
|  |  |
